# A reconstruction of sexual modes throughout animal evolution

**DOI:** 10.1186/s12862-017-1071-3

**Published:** 2017-12-06

**Authors:** Daniel A. Sasson, Joseph F. Ryan

**Affiliations:** 1 Whitney Laboratory for Marine Bioscience, University of Florida, 9505 Ocean Shore Blvd, St. Augustine, FL USA; 2 Department of Biology, University of Florida, Gainesville, FL USA

**Keywords:** Hermaphroditism, Gonochorism, Ancestral state reconstruction, Sexual mode, Reproduction

## Abstract

**Background:**

Although most extant animals have separate sexes, simultaneous hermaphrodites can be found in lineages throughout the animal kingdom. However, the sexual modes of key ancestral nodes including the last common ancestor (LCA) of all animals remain unclear. Without these data, it is difficult to infer the reproductive-state transitions that occurred early in animal evolution, and thus a broad understanding of the evolution of animal reproduction remains elusive. In this study, we use a composite phylogeny from four previously published studies, two alternative topologies (ctenophores or sponges as sister to the rest of animals), and multiple phylogenetic approaches to conduct the most extensive analysis to date of the evolution of animal sexual modes.

**Results:**

Our analyses clarify the sexual mode of many ancestral animal nodes and allow for sound inferences of modal transitions that have occurred in animal history. Our results also indicate that the transition from separate sexes to hermaphroditism has been more common in animal history than the reverse.

**Conclusions:**

These results provide the most complete view of the evolution of animal sexual modes to date and provide a framework for future inquiries into the correlation of these transitions with genes, behaviors, and physiology. These results also suggest that mutations promoting hermaphroditism have historically been more likely to invade gonochoristic populations than vice versa.

**Electronic supplementary material:**

The online version of this article (10.1186/s12862-017-1071-3) contains supplementary material, which is available to authorized users.

## Background

How and why animals transition between simultaneously hermaphroditic (concurrently male and female) and gonochoristic (separate sexes) sexual modes are among the most fascinating questions in animal evolution. While the majority of animals are gonochoristic, hermaphroditism is estimated to occur in 5–6% of animal species (and almost one-third of non-insect species), with over 70% of animal phyla containing at least one hermaphroditic species [1]. Given the phylogenetic spread of these characters, it is likely that the evolution of gonochorism from hermaphroditism or vice versa has occurred many times throughout evolution. However, only a very few studies have attempted to identify these transitions e.g., [2], and the ancestral sexual mode of many major animal lineages remains unclear. These transitions are important evolutionary events with the condition of separate sexes giving rise to some of the most elaborate morphological and behavioral traits in animals [3] and simultaneous hermaphroditism leading to a range of unique behavioral and physiological adaptions e.g., [4–6].

The selective pressures that lead to transitions from gonochorism to hermaphroditism or vice versa in animals are unclear. In flowering plants, separate sexes are primarily thought to be an adaptation to avoid inbreeding due to self-fertilization e.g., [7–9]; the benefits of reproductive assurance and increased genetic relatedness to offspring are traditionally proposed to explain the transition from separate sexes to self-compatible hermaphrodites e.g., [7], although transitions in this direction are rare [10]. Avoidance of inbreeding depression has been less emphasized as a driving force for the transition from hermaphroditism to gonochorism in animals than in plants [11], possibly because many simultaneously hermaphroditic animals, especially those with internal fertilization, are obligate out-crossers [9]; however, inbreeding depression has been shown to occur in some self-fertile animals e.g., [12–16]. Much of the theoretical work explaining when separate sexes should be favored over simultaneous hermaphroditism has focused on resource allocation trade-offs between male and female functions; that is, separate sexes will be favored when increased investment in one sex provides higher fitness than investment in both sexes e.g., [17]. Conversely, self-fertile hermaphroditism is predicted to be advantageous in systems where mating partners are infrequent and/or mate search ability is low, thereby providing reproduction assurance or increasing the odds of finding a compatible partner [1, 11, 18–20]. A recent study found support for this hypothesis; mate search ability and transitions between sexual modes are correlated across multicellular organisms [21]. Additionally, hermaphroditism may be optimal when fitness gains from allocating resources into one sexual function reach a saturation point (i.e., little reproductive gain for increased allocation to that sex) [22], although the empirical evidence for such saturation curves in simultaneous hermaphrodites is scant [23]. In order to evaluate theoretical models related to the underlying factors that have governed sexual mode transitions in animals throughout evolution, it is important to broaden the reconstruction of ancestral states across the animal tree of life.

Speculation on the ancestral sexual mode of multicellular organisms has been frequent since at least Darwin [24]. However, a majority of theoretical and empirical research into the transitions between hermaphroditism and separate sexes has focused on plant systems e.g., [8, 24–26]. In flowering plants, self-fertilizing hermaphroditism is believed to have derived from out-crossing hermaphroditic species e.g., [27, 28] with the reverse transition being rare e.g., [29] . Furthermore, separate sexes are uncommon in flowering plant species and believed to be the derived condition e.g., [7, 30].

Unlike in plants, there has been little agreement about the ancestral sexual mode of major animal lineages despite over a hundred years of debate on the topic. Pelseneer [31] concluded that gonochorism is the ancestral state of animals based on a detailed study of molluscs. Conversely, Ghiselin [18] proposed that hermaphroditism is generally derived in animals and proposed a number of models to explain its presence. Only a few studies have examined in detail the transitions between gonochorism and hermaphroditism in animals e.g., [2] and thus whether there exists a bias in the direction of these transitions is unclear. Recent studies have used ancestral state reconstruction methods to tackle these questions; however, these results have proven equivocal. Iyer & Roughgarden [32] concluded that the last common ancestor (LCA) of all animals was likely hermaphroditic and that transitions from hermaphroditism to gonochorism were more frequent in evolution than the reverse. However, these conclusions were based on a tree that included a paraphyletic Spiralia, codings of sexual mode at the phyla level rather than the species level, and methods exclusively based on maximum parsimony. Conversely, Eppley & Jesson [21] showed equal support for hermaphroditism and gonochorism in the LCA of animals and suggested that transitions from separate sexes to hermaphroditism were more likely across multicellular organisms. However, this tree included a polyphyletic Porifera and, like Iyer & Roughgarden [32], this study used phylum- or class-level codings rather than at the level of species, which only allows for the identification of transitions at a broad scale. Finally, neither Iyer & Roughgardern [32] nor Eppley & Jesson [21] incorporated branch lengths into their analyses (i.e. all branch lengths were considered equal), which are important components for estimating ancestral states. Since the publication of these studies, large-scale data sets, greater taxon sampling, and phylogenomic methodologies have altered the phylogenetic relationships of several animal lineages that are key to our understanding of early animal evolution and have provided a rich set of data to apply to this question e.g., [33–43].

In this study, we apply broad taxon sampling, multiple topologies, and several phylogenetic approaches in the most extensive effort to date to reconstruct the ancestral sexual modes of a wide range of animal ancestors and to identify transitions of sexual mode in animal evolution. Our work provides a more sophisticated understanding of how sexual mode has evolved in animals and a basis for detailed investigations into the selective pressures and genetic changes underlying these extraordinary evolutionary events.

## Methods

### Tree topology

We manually constructed a composite phylogeny based on four previously published studies. We used Cannon et al. [44] as the backbone topology of the animal tree and for all of the specific taxa within Bilateria. To increase sampling from non-bilaterian lineages, we incorporated the full topologies from recent phylogenies of three groups – Ctenophora [45], Porifera [46], and Cnidaria [47] – into the Cannon et al. [44] topology. Riesgo et al. [46] include both a polyphyletic and monophyletic sponge topology in their study; we selected the monophyletic topology for our composite tree, which is consistent with the majority of phylogenomic studies including Cannon et al. [44].

### Branch lengths

Branch lengths provide information about the amount of evolutionary change along each lineage. Stochastic character mapping approaches to ancestral state reconstruction analyses incorporate branch lengths when predicting ancestral states. Our composite tree incorporated trees from distinct phylogenetic studies. The branch lengths from each of these trees were not compatible with each other; therefore, we performed a maximum-likelihood analysis using an alignment of 18S sequences from the taxa in our composite tree under a topology constrained to be congruent with the composite tree to estimate branch lengths. When we could not find at least a partial 18S rRNA sequence for a species (N = 6), we substituted that species in our tree for another member of the same genus whose 18S sequence was readily available online. In the case of *Halitrephes valdiviae*, we could not find an 18S sequence for any species within the same genus and so we replaced it with *Botrynema brucei*, which is in the same family. In the case of *Nephthyigorgia sp*. we could not find an 18S sequence within the same family. Here we substituted a sequence from *Dendronephthya putteri* (Additional file 1: Table S1), which is in the same suborder.

We aligned the 18S sequences using SSU-Align version 0.1.1 [48] with default parameters. We converted the output of SSU-Align to FASTA format. We then trimmed the resulting alignment with Gblocks version 0.91b [49] using dynamic parameters generated by Gblockswrapper version 0.03 (https://goo.gl/fDjan6). We converted the output of Gblocks to Phylip format and then calculated branch lengths with RAxML version 8.1.21.

To test whether our branch lengths based on a single gene (i.e. 18S) were comparable to those based on many genes, we used Spearman’s rank correlation test to compare our Bilateria and Porifera branch lengths to those found in Cannon et al. [44] and Riesgo et al. [46], respectively.

### Ancestral state reconstruction

We characterized the reproductive state for each animal species in our tree (N=167; Additional file 1: Table S1) based on information obtained through detailed literature searches. We removed choanoflagellate outgroups from the ancestral state reconstruction since it is very difficult to match sexual mode in a unicellular organism to that of a multicellular organism. For the purposes of this study, we characterized species as gonochoristic if they have separate sexes at any point during their sexually active life-cycle; under this framework we consider sequentially hermaphroditic animals as gonochoristic as has been done in other ancestral state reconstructions [2, 21, 32]. We coded animal species as unknown in cases where we could not find a description of the sexual mode for that species or another member of the genus (N = 25). A number of cnidarians have zooids of multiple sexes within one colony [50, 51]. We classified species as gonochoristic if these colonies bud separate sexed reproductive medusae (i.e., *Clytia hemisphaerica* [52]). However, in some siphonophores, the reproductive medusae (gonophores) remain as part of the colonies. We classified three species that have gonophores of both sexes within one colony as hermaphroditic (*Ablylopsis tetragona*, *Agalma elegans*, and *Nanomia bijunga*) [50, 51]. We found contradictory reports for the sexual mode of *Membranipora membranacae* [53, 54]; therefore, we coded this species as unknown. The species *Anemonia viridis* and *Hydra magnipapillata* reproduce both sexually and asexually e.g., [55, 56]; we coded these species as hermaphroditic and gonochoristic, respectively. Finally, three species (*Adineta ricciae, Adineta vaga*, and *Lepidermella squamata*) reproduce primarily through asexual reproduction and we coded them as such. We characterized all other species as either hermaphroditic or gonochoristic (see Additional file 1: Table S1 for details).

We used Mesquite v3.04 [57] to perform maximum-parsimony ancestral state reconstructions. We used an unordered states assumption for our parsimony models [58]. Default settings were used for all other parameters. We also used Mesquite to count transitions between sexual modes for the maximum-parsimony analyses. Since Mesquite produced multiple equally parsimonious trees, the number of transitions for the parsimony analysis was averaged across these trees.

We also used stochastic character mapping [58] to infer sexual mode using R (v3.2.0). We first used the ace function in the ape (v4.1) package [59] to estimate ancestral character states using a one-parameter equal-rates model (ER), a three-parameter symmetrical model (SYM), and a six-parameter all-rates-different model (ARD). We then compared the fit of these models to these data by performing a likelihood ratio test on the resulting –ln L scores ((1-pchisq(Δ − ln L, df)). We applied the make.simmap function from the phytools package (v0.6.20) [60] using the model with the best fit and 1000 simulations. The R scripts, data files, and outputs for both of these analyses are included in the GitHub repository that accompanies this paper.

In the maximum-parsimony analyses, taxa with undetermined sexual modes were coded with an unknown character state. In the stochastic character mapping analyses, the function make.simmap calculates a likelihood for the sexual mode at the tips with an unknown character state during the ancestral state reconstruction.

There remains debate as to whether ctenophores e.g., [33–40] or sponges e.g., [41–43] are the sister taxon to the rest of animals. For this reason, we ran all ancestral state reconstruction analyses with both “ctenophore-sister” and “sponge-sister” topologies.

### Effect of branch lengths

Recent studies reconstructing the ancestral sexual state of animals did not take branch length into account (21, 23). To estimate the effect of branch lengths on our analyses, we also ran a stochastic character mapping analysis with all branches set to equal lengths.

## Results

We manually created a composite tree from four previously published studies [44–47]. In total, this composite tree consisted of 165 animal species and two choanoflagellete outgroups; most of the major animal lineages were well represented in the tree, with 62 sponges, 60 bilaterians, 31 cnidarians, 11 ctenophores, and one placozoan. Our final set included 83 gonochoristic, 54 hermaphroditic, three asexual, and 25 uncharacterized animal species (cases where we could not find a definitive source on their sexual mode) (Additional file 1: Table S1).

### Branch lengths

We compared the branch lengths we generated (Additional file 2: Figures S1, Additional file 3: Figure S2) using 18S to the branch lengths found for Porifera in Riesgo et al. [46] and Bilateria in Cannon et al. [44], which were generated using two and 212 gene alignments, respectively. Our branch lengths were significantly correlated to both the branch lengths generated for Porifera by Riesgo et al. [46] (N = 62, Spearman’s rank correlation test, ρ = 0.79, p < 0.0001) and for Bilateria by Cannon et al. [44] (N = 60, Spearman’s rank correlation test, ρ = 0.60, *p* < 0.0001). Our composite trees with branch lengths can be seen in the supplemental information (Additional file 2: Figure S1, Additional file 3: Figure S2).

### Maximum parsimony ancestral state reconstructions

**Fig. 1:**
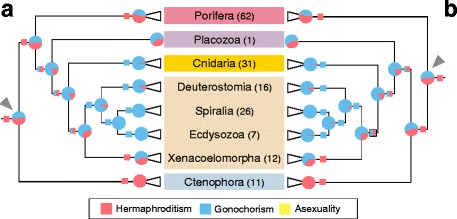
Ancestral-state reconstruction of sexual modes in animals for both (**a**) “ctenophore-sister” and (**b**) “sponge-sister” topologies. For each cladogram, the darkened line indicates the sister lineage to the rest of animals. Numbers in parentheses display the number of animals in each lineage used in the analyses. Circles at nodes depict the states assigned by stochastic character mapping while boxes display the maximum parsimony-assigned states. Blue represents gonochorism, red represents hermaphroditism, and yellow represents asexuality. Boxes evenly divided between red and blue colors indicate that gonochorism and hermaphroditism are equally parsimonious explanations of the data. The grey arrows point to the nodes representing the most recent common ancestor of extant animals. Major clades have been collapsed in this figure. The uncollapsed versions are available as Additional file 4: Figures S3, Additional file 5: Figures S4, Additional file 6: Figures S5, Additional file 7: Figures S6

We used two distinct topologies in our ancestral state analyses; the one consistent with the Cannon et al., [44] backbone, which had Ctenophora as the sister group to the rest of animals (“ctenophore-sister”) and the other, which situated Porifera as the sister group to the rest of animals (“sponge-sister”). The maximum-parsimony analyses were consistent regardless of whether the analyses were performed on the “ctenophore-sister” (Fig. 1a; Additional file 4: Figure S3) or “sponge-sister” trees (Fig. 1b; Additional file 5: Figure S4). Hermaphroditism was the most parsimonious sexual mode for the LCA of ctenophores, the LCA of sponges, and the LCA of all animals (Fig. 1). Gonochorism and hermaphroditism were equally well supported in the cnidarian-bilaterian LCA and the bilaterian LCA. Lastly, the LCA of Cnidaria and the LCA of Nephrozoa (i.e., all Bilateria except Xenoturbella and Acoelomorpha) were both designated as gonochoristic (Fig. 1).

### Stochastic character mapping reconstruction of ancestral states

A comparison of the log likelihoods of the three models available to the stochastic character mapping implementation in phytools supported the use of the SYM model in both the ctenophore-sister and sponge-sister analyses. The SYM model fit the data significantly better than the ER model (χ^2^ = 54.9, df = 2, p < 0.0001) but not significantly better than the ARD model (χ^2^ = 2.6, df = 3, p = 0.45) for the “ctenophore-sister” tree. We found a similar result for the sponge-sister tree as well ( χ^2^ = 54.2, df = 2, p <0.0001 for SYM vs. ER models and χ^2^ = 1.9, df = 3, p = 0.59 for the SYM vs. ARD models). The SYM model uses fewer parameters than the ARD model and since the two models fit the data equally well, the SYM model was used for stochastic character mapping analyses.

**Fig. 2:**
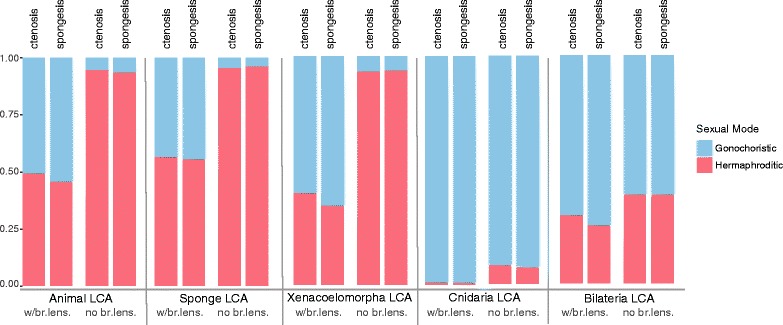
Comparison of selected nodes on stochastic character mapping trees with and without estimated branch lengths. For each node (e.g., Animal LCA) the ctenophore-sister and sponge-sister percentages are represented for the analyses with estimated branch lengths and the ones with no branch lengths (i.e. equal branch lengths). Asexual is not represented here because it was 0.00 at each of these nodes. ctenosis = ctenophore sister; spongesis = sponge sister; LCA = last common ancestor; br.lens. = branch lengths

The ancestral states resulting from our stochastic character mapping analyses using the “ctenophore-sister” (Fig. 1a) and “sponge-sister” (Fig. 1b) topologies were mostly in agreement (Fig. 1 & 2; Additional file 6: Figure S5, Additional file 7: Figure S6; Additional file 8: Table S2). Both the “ctenophore-sister” and “sponge-sister” analyses favored a hermaphroditic LCA for Poriferia and Ctenophora and a gonochoristic LCA for Cnidaria and Bilateria (Fig. 1; Additional file 8: Table S2). All nodes within Bilateria favored separate sexes, although the LCA of Xenacoelomorpha was more equivocal. Unlike the maximum-parsimony analyses, however, both the “ctenophore-sister” and “sponge-sister” analyses slightly favored gonochorism as the sexual mode of the last common ancestor of all animals (51% and 54% for “ctenophore sister” and “sponge sister” respectively, Fig. 1).

Previous recent studies applied likelihood-based methods to the origin of sexual mode, but without including variable branch lengths (21, 23). To test the effect of branch-lengths we ran our analyses with the SYM model using equal branch lengths. These analyses resulted in a very different estimate for the sexual mode of the ancestor of several deep ancestral nodes (Fig. 2, Additional file 9: Figure S7 & Additional file 10: Figure S8). For example, hermaphroditism was overwhelmingly predicted to be the sexual state of the LCA of sponges, xenacoelomorphs, and all animals in the equal-branch length analyses (Fig. 1), but not with estimated branch lengths (Fig. 1). Some nodes like the Cnidaria and Bilateria LCA were much less affected (Fig. 2).

### Transitions between sexual modes

Both the maximum-parsimony and stochastic character mapping analyses had more transitions from gonochorism to hermaphroditism than vice versa (Table 1). Averaged across equally maximum-parsimonious trees, we found 13.58 and 13.18 transitions from gonochorism to hermaphroditism, and 6.42 and 6.82 transitions from hermaphroditism to gonochorism in our “ctenophore-sister” and “sponge-sister” trees, respectively. Unlike parsimony, stochastic character mapping allows for more than one transition per branch. Across the 1000 simulated trees of the stochastic character mapping analyses, we found an average of 48.1 and 48.3 transitions from gonochorism to hermaphroditism and 32.7 and 32.5 transitions from hermaphroditism to gonochorism in our “ctenophore-sister” analysis and “sponge-sister” topologies, respectively. In order to visualize where transitions occur in our stochastic character mapping analyses, we approximated the position of transitions along branches by plotting all 1000 simulations using the densityTree function of phytools (Additional file 11: Figure S9 & Additional file 12: Figure S10).

**Table 1:** Average changes across 1000 replicates of stochastic character mapping. The first value was estimated on the ctenophore sister topology (Fig. 1a) and the second (separated by ‘/’) was estimated on the sponge sister topology (Fig. 2a)

	Hermaphrodite	Separate sexes	Asexual
Hermaphrodite	-	32.713 / 32.505	0 / 0
Separate sexes	48.09 / 48.254	-	2.236 / 2.25
Asexual	0 / 0	0.07 / 0.85	-

## Discussion

In this study, we assembled a 165-taxon tree and determined the sexual mode for the majority of these taxa in order to infer ancestral sexual modes of many key positions across the animal tree of life. By doing so, we identified transitions between sexual modes throughout animal evolutionary history. Across all analyses, we recovered few differences between inferences made on the “ctenophore-sister” and “sponge-sister” topologies, although in general the “sponge-sister” topology provided a slightly higher likelihood of gonochorism for the LCA of a number of nodes (Figs. 1 & 2, Additional file 8: Table S2). Overall, however, the results suggest that the placement of these early lineages does not greatly influence the predicted sexual modes or the number of transitions between states in animal evolution.

Our stochastic character mapping analyses provide strong predictions of the sexual mode for most of the deep ancestral nodes within animals. In all of our analyses we found robust support for the LCA of ctenophores as hermaphroditic (Figure 1, Additional file 8: Table S2). This is not surprising given that only three of the more than 150 described ctenophore species have been shown to have separate sexes [61, 62]. Regardless of topology, the maximum-parsimony and stochastic character mapping reconstructions favored hermaphroditism as the condition of the LCA of sponges (Fig. 1; Additional file 8: Table S2). Interestingly, these results contrast with the conclusions of Riesgo et al. [46] that slightly favored gonochorism for the sponge LCA. This difference between these studies shows the effect that increased taxon sampling outside of the clade of interest can have on the estimated sexual mode of that clade’s LCA.

While the ancestral sexual mode of the cnidarian/bilaterian ancestor remains unresolved in our parsimony analyses, our stochastic character mapping analyses predict that this ancestor was likely gonochoristic. Additionally, we see strong support for gonochorism as the ancestral state of the cnidarian LCA. Cnidarian hermaphroditism seems to be mainly confined to the Hexacorallia and Siphonophora (Additional file 4: Figure S3, Additional file 5: Figure S4, Additional file 6: Figure S5, Additional file 7: Figure S6), and within these groups hermaphroditism is fairly common [50, 51, 63]. The sexual mode of the LCA of Bilateria remains unresolved in both our parsimony analyses while the stochastic character mapping analyses predicts a relatively high likelihood of gonochorism (Fig. 1, Additional file 8: Table S2).

Our results concerning the identity of the LCA of all animals are mixed (Fig. 1, Additional file 8: Table S2). The analyses incorporating “ctenophore-sister” and “sponge-sister” topologies were largely in agreement; in both cases, our stochastic character mapping approach found equivocal support for the sexual mode of the last common ancestor (LCA) of all animals. Conversely, both parsimony analyses supported the hypothesis that the LCA was hermaphroditic. The uncertainty of our stochastic character mapping result may be somewhat surprising considering the strong support for hermaphroditism for the LCAs of ctenophores and the more likely than not hermaphroditism for the LCA of sponges since these clades represent the two earliest branching animal lineages. It could be that the overwhelming support for a gonochoristic cnidarian-bilaterian LCA strongly influences the animal LCA.

Taxa at key phylogenetic positions heavily influence the stochastic character mapping ancestral-reconstruction predictions for deeper nodes. For example, Xenacoelomorpha is the sister to the rest of Bilateria, and *Xenoturbella bocki*, a hermaphrodite, is the sister to the remaining Xenacoelomorpha. However, the only other *Xenoturbella* species whose sexual mode has been described, *X. profunda*, is gonochoristic [64]. Replacing *X. bocki* with *X. profunda* has an especially profound effect on the reconstructed sexual mode of the LCA of Bilateria, which in turn influences the reconstructed sexual mode of the bilaterian-cnidarian LCA (Additional file 13: Figure S11 & Additional file 14: Figure S12). The sexual mode of the other members of *Xenoturbella* remains undetermined [64] and so whether hermaphroditism or gonochorism is the prevalent sexual mode in that lineage is currently unknown. This example reinforces the widely held view that the number and choice of taxa included in ancestral-state reconstruction analyses have a profound influence on the results. To avoid selection bias and to provide an objective criterion for taxon inclusion, we limited the species in our analyses to those from the papers upon which we based our tree (with a few exceptions due to availability of 18S sequences, see Methods and Additional file 2: Figure S1). Having an objective *a priori* reason for the inclusion of specific taxa for analysis is essential in ancestral state reconstructions. Nevertheless, the taxa used in our analysis are not always the most representative examples of sexual mode in their respective clades, which may negatively affect our inferences. The soundness of our method of taxon sampling will increase as phylogenomic studies expand and the effects of a few unrepresentative taxa diminish.

Our ancestral-state reconstruction results are greatly influenced by the node-to-tip branch lengths throughout the tree. We used a single gene (18S) to estimate branch lengths across our taxa. Our estimated branch lengths correlated well with the branch lengths estimated from two genes in sponges [46] and relatively well with branch lengths generated from hundreds of genes [44]. Branch length estimations can have a profound impact on ancestral-state reconstruction results; for example, setting all branch lengths equal results in hermaphroditism being overwhelmingly predicted as the sexual mode of the LCA of all animals (Fig. 2; Additional file 9: Figure S7 & Additional file 10: Figure S8). In the near future, the availability of large phylogenomic datasets with extensive taxon sampling across all animal lineages will allow for more accurate branch length estimates and thus more precise ancestral state reconstructions.

We found more transitions from gonochorism to hermaphroditism than the reverse in both our stochastic character mapping and parsimony ancestral-state analyses (Table 1). These results may seem somewhat counter-intuitive as the genetics of a gonochoristic animal gaining a sexual function would seem more complicated than the loss of a sexual function in a hermaphroditic animal [2], yet a similar conclusion has been found in other studies [2, 18, 21, but see 32]. These results may indicate that factors promoting hermaphroditism in gonochoristic animals (e.g., reproductive assurance) are more widespread or create stronger selection pressures than the conditions promoting gonochorism in hermaphroditic systems (e.g., inbreeding depression). Inbreeding depression in particular may not be a strong selective factor in many simultaneous hermaphrodites with internal fertilization where self-fertilization is unlikely [9]. Interestingly, this trend is the reverse to what is seen in flowering plants, where the avoidance of inbreeding depression has led to a higher number of transitions from hermaphroditism to separate sexes than the reverse e.g., [7–9].

Many transitions between hermaphroditism and gonochorism in both plants and animals almost certainly involve intermediate reproductive states (e.g., androdioecy) where not all individuals of a system are simultaneous hermaphrodites or gonochorists (reviewed in [2]). We coded all sexually reproducing taxa as either simultaneous hermaphrodites or gonochoristic, but many animal species have more complicated sexual modes, such as sequential hermaphroditism. We classified sequential hermaphrodites as gonochoristic like in [2, 21, 32]. Our reasoning is that sequential hermaphrodites functionally act as only a single sex at a given time and thus selection related to sexual mode (e.g., inbreeding depression, mate search ability, etc.) has more in common with gonochoristic systems. However, the genetics underlying sequential vs. simultaneous hermaphroditism are not well understood. It could be that these two are more similar genetically and should be considered differently in ancestral-state reconstructions. It may be possible to apply a more complex model to future analyses, which better integrates the subtle relationships between separate sexes, simultaneous hermaphroditism, and sequential hermaphroditism.

Many of the transitions to hermaphroditism found in our analyses occurred in Cnidaria and Porifera suggesting that these lineages may be particularly conducive to hermaphroditism. In Cnidaria, other than tube anemones, all of the hermaphroditic species are colonial. Colonial hermaphroditism is somewhat of a special case of hermaphroditism since in most examples individual zooids are separate sexes, but each sex is represented on the colony [51]. As such, it may be easier for colonial organisms to transition from separate sexes to hermaphroditism.

In Cnidaria as well as Porifera, other than the pelagic Siphonophora, the hermaphroditic species are mostly sessile as adults, which perhaps supports the hypothesis that movement ability and hermaphroditism are correlated e.g., [21], but this intepretation should be taken with caution since many sessile taxa within these lineages have separate sexes. It should be noted, however, that while many transitions are found in Porifera and Cnidaria, these are the two best-sampled clades in our analyses. High numbers of transitions would certainly have been observed in some other clades, such as Platyhelminthes [65] or fish [66], if they had been similarly represented. We do still see a few transitions from gonochorism to hermaphroditism within Biliateria. Hermaphroditism within Bilateria was independently derived and most widespread in Xenacoelomorpha and Platyhelminthes. Both groups share several morphological traits with each other (and cnidarians, including ciliated epidermis and soft body forms); with more taxon sampling, it could be insightful to look at these (and other) traits across animals to see if they correlate with tendencies to switch sexual modes.

Our analyses have also identified a number of transition points in shallow nodes that could be potentially fruitful for more in-depth investigation. For example, within Bilateria, our analysis has inferred a transition from hermaphroditism to gonochrorism after the *Schistostoma mansoni* lineage split from its shared LCA with *Taenia pisiformes* (Additional file 2: Figure S1). We see a similar transition within ctenophores; gonochorism evolved after *Ocyropsis maculata* and *Velamen parallelum* split. Interestingly, members of the *Ocyropsis* genus have a greater ability for directed movement than other ctenophore species, providing another example of how movement ability and sexual mode may be linked e.g., [65]. A comparison of other behavioral, morphological, or genetic differences between sister taxa may prove insightful in the above cases.

## Conclusions

This study represents the most extensive attempt thus far to reconstruct the ancestral sexual mode of key ancestral nodes within animals. Our models have identified key transitional nodes throughout the animal tree and clarified the ancestral sexual mode for a number of lineages. Despite our increased taxon sampling, there are still several key ancestral nodes that remain unresolved including the LCA of all animals. As increasing numbers of dense phylogenomic studies of animal clades are published, it will be possible to greatly expand the taxon sampling using the composite approach outlined in this study. Repeating these analyses with expanded taxa and further phylogenetic clarifications will lead to still greater resolution to the ancestral states of sexual mode in animals.

Our results can now be used as a backbone to test for correlated evolution between behavioral, morphological, and life-history traits hypothesized to be linked to the transitions between gonochorism and hermaphroditism. Furthermore, the identification of transitional nodes will open the door for comparative genomic approaches to detect the genetic architecture underlying these transitions. These types of analyses will become even more tenable as future studies with increased taxon sampling locate additional clades with high numbers of transitions. Moreover, the topology and composite approach outlined in this study may be useful as a template to reconstruct the ancestral state of other animal traits. Finally, as with early phylogenetic studies of animal relationships, these results will set the bar for future progress in the field regardless of whether they agree with future data-rich studies.

## Additional files

Additional file 1: Table S1. Sexual mode and sequence information for included taxa. Accession/gi numbers are from GenBank. H = simultaneous hermaphrodite, S=separate sexes (or sequential hermaphrodite), A = Asexual, ? = unknown. Likelihood values from our stochastic character mapping analyses for the predicted sexual mode of the LCA of major animal lineages. (DOCX 43 kb).

Additional file 2: Figure S1. Composite “ctenophore-sister” tree showing branch lengths generated from the use of one gene (18S). Figure generated using FigTree (http://tree.bio.ed.ac.uk/software/figtree/). (PDF 13 kb)

Additional file 3: Figure S2. Composite “sponge-sister” tree showing branch lengths generated from the use of one gene (18S). Figure generated using FigTree (http://tree.bio.ed.ac.uk/software/figtree/). (PDF 9 kb)

Additional file 4: Figure S3. Full cladogram of maximum-parsimony ancestral-state reconstruction of sexual mode for the “ctenophore-sister” topology using Mesquite. The color blue represents gonochorism, red represents hermaphroditism, yellow represents asexuality, and grey represents an unknown sexual mode. (PDF 78 kb)

Additional file 5: Figure S4. Full cladogram of maximum-parsimony ancestral-state reconstruction of sexual mode for the “sponge-sister” topology using Mesquite. The color blue represents gonochorism, red represents hermaphroditism, yellow represents asexuality, and grey represents an unknown sexual mode. (PDF 78 kb)

Additional file 6: Figure S5. Ancestral reconstruction of sexual mode on the “ctenophore-sister” topology from stochastic character mapping. The color blue represents gonochorism, red represents hermaphroditism, and yellow represents asexuality. We used a symmetrical rates model in the make_simmap function from the phytools package. Tree was converted to ultrametric using the ‘*chronopl*’ command in the Ape package. R script used to generate this PDF is available here: https://github.com/josephryan/2017b_Sasson_and_Ryan. (PDF 139 kb)

Additional file 7: Figure S6. Ancestral reconstruction of sexual mode on the “sponge-sister” topology from stochastic character mapping. The color blue represents gonochorism, red represents hermaphroditism, and yellow represents asexuality. We used a symmetrical rates model in the make_simmap function from the phytools package. Tree was converted to ultrametric using the ‘*chronopl*’ command in the Ape package. R script used to generate this PDF is available here: https://github.com/josephryan/2017b_Sasson_and_Ryan. (PDF 135 kb)

Additional file 8: table S2. Likelihood values from our stochastic character mapping analyses for the predicted sexual mode of the LCA of major animal lineages. (DOCX 18 kb).

Additional file 9: Figure S7. Effect of branch lengths shown through ancestral state reconstruction of sexual mode on the “ctenophore-sister” topology without branch lengths. This analysis is the same as Additional file 6: Figure S5 except branch lengths were equal in this analysis. R script used to generate this PDF is available here: https://github.com/josephryan/2017b_Sasson_and_Ryan. (PDF 187 kb)

Additional file 10: Figure S8. Effect of branch lengths shown through ancestral state reconstruction of sexual mode on the “sponge-sister” topology with equal branch lengths. This analysis same as Additional file 7: Figure S6 except branch lengths were equalized in this the analysis. R script used to generate this PDF is available here: https://github.com/josephryan/2017b_Sasson_and_Ryan. (PDF 190 kb)

Additional file 11: Figure S9. Transitions across the stochastic character mapping ancestral state reconstruction of the “ctenophore-sister” topology averaged over 1000 simulations. Blue represents separate sexes, red hermaphroditism, and yellow asexuality. Gradations between those colors indicate areas where transitions were simulated to have occurred. R script used to generate this PDF is available here: https://github.com/josephryan/2017b_Sasson_and_Ryan. (PDF 64 kb)

Additional file 12: Figure S10. Transitions across the stochastic character mapping ancestral state reconstruction of the “sponge-sister” topology averaged over 1000 simulations. Blue represents separate sexes, red hermaphroditism, and yellow asexuality. Gradations between those colors indicate areas where transitions were simulated to have occurred. R script used to generate this PDF is available here: https://github.com/josephryan/2017b_Sasson_and_Ryan. (PDF 4 kb)

Additional file 13: Figure S11. Stochastic character mapping ancestral-state reconstruction for the “ctenophore-sister” topology with the gonochrositc *Xenoturbella profunda* replacing the hermaphrodite *X. bocki*. The inclusion of the gonochoristic *X. profunda* has an especially strong effect on the LCA of Bilateria. Blue circles indicate gonochorism, red circles hermaphroditism, and yellow circles asexuality. (PDF 157 kb)

Additional file 14: Figure S12. Stochastic character mapping ancestral-state reconstruction for the “sponge-sister” topology with the gonochrositc *Xenoturbella profunda* replacing the hermaphrodite *X. bocki*. The inclusion of the gonochoristic *X. profunda* has an especially strong effect on the LCA of Bilateria. Blue circles indicate gonochorism, red circles hermaphroditism, and yellow circles asexuality. (PDF 157 kb)

## Abbreviations

LCA: Last common ancestor
