# Supplementary material for: A reconstruction of sexual modes throughout animal evolution
Source: BMC Evol Biol. 2017 Dec 6;17:242. doi: 10.1186/s12862-017-1071-3 (PMC5717846; doi:10.1186/s12862-017-1071-3)
Supplement: Supplementary file 1 — Sexual mode and sequence information for included taxa. Accession/gi numbers are from GenBank. H = simultaneous hermaphrodite, S=separate sexes (or sequential hermaphrodite), A = Asexual, ? = unknown. Likelihood values from our stochastic character mapping analyses for the predicted sexual mode of the LCA of major animal lineages. (DOCX 43 kb). [file 12862_2017_1071_MOESM1_ESM.docx]

.

**Additional file 1: Table S1.** Sexual mode and sequence information for included taxa. Accession/gi numbers are from GenBank. H = simultaneous hermaphrodite, S=separate sexes (or sequential hermaphrodite), A = Asexual, ? = unknown.

| **Lineage** | **Species** | **Sexual**  **mode** | **Reference for**  **sexual mode** | **Accession/gi** |
| --- | --- | --- | --- | --- |
| Choanoflagellata | *Monosiga*  *brevicollis* | ? |  | AF084618 |
| Choanoflagellata | *Proterospongia*  *sp.* | ? |  | EU011924 |
| Cnidaria | *Abylopsis*  *tetragona* | H | Dunn et al. 2005 | AY937345 |
| Cnidaria | *Acropora*  *longicyathus* | H | Kerr et al. 2011 | AF038031 |
| Cnidaria | *Acropora*  *millepora* | H | Kerr et al. 2011 | AF038032 |
| Cnidaria | *Aegina*  *citrea* | ? |  | AF358058 |
| Cnidaria | *Agalma*  *elegans* | H | Dunn et al. 2005 | AY937313 |
| Cnidaria | *Alatina*  *mordens* | S | Kingsford & Mooney 2014 | GQ849082 |
| Cnidaria | *Alcyonium*  *gracillimum* | S | Hwang & Song 2009 | JQ688007 |
| Cnidaria | *Anemonia*  *viridis* | S | Shick 1991 | EU190849 |
| Cnidaria | *Anthomastus*  *sp.* | S | Cordes et al. 2001 | AF052881 |
| Cnidaria | *Antipathes*  *griggi* | S | Wagner et al. 2012 | FJ389904 |
| Cnidaria | *Atolla*  *vanhoeffeni* | S | Lucas & Reed 2010 | AF100942 |
| Cnidaria | *Aurelia*  *aurita* | S | Lucas 2001 | AY039208 |
| Cnidaria | *Botrynema*  *brucei* | ? |  | EU247822 |
| Cnidaria | *Candelabrum*  *cocksii* | S | Prada & Hellberg 2013 | AY920758 |
| Cnidaria | *Cerianthus*  *borealis* | H | Hinsch & Moore 1992 | AF052897 |
| Cnidaria | *Clytia*  *hemisphaerica* | S | Carré & Carré 2000 | AY789753 |
| Cnidaria | *Craseoa*  *lathetica* | H | Dunn et al. 2005 | AY937339 |
| Cnidaria | *Dendronephthya*  *putteri* | S | Hwang & Song 2007 | AJ133550 |
| Cnidaria | *Ectopleura*  *larynx* | S | Nawrocki 2012 | AY920760 |
| Cnidaria | *Exaiptasia*  *pallida* | S | Grawunder et al. 2015 | KP761308 |
| Cnidaria | *Haliclystus*  *sanjuanensis* | S | Eckelbarger & Larson 1993 | AF358102 |
| Cnidaria | *Hydra*  *magnipapillata* | S | Sugiyama & Fujisawa 1977 | EF059942 |
| Cnidaria | *Hydractinia symbiolongicarpus* | S | Grosberg et al. 1996 | EU272621 |
| Cnidaria | *Keratoisidinae*  *sp.* | ? |  | FJ358837 |
| Cnidaria | *Metridium*  *senile* | S | Kaplan 1983 | JF832982 |
| Cnidaria | *Montastraea*  *annularis* | H | Levitan et al. 2004 | AF238267 |
| Cnidaria | *Nanomia*  *bijuga* | H | Dunn et al. 2005 | AF358071 |
| Cnidaria | *Nematostella*  *vectensis* | S | Hand & Uhlinger 1992 | AF254382 |
| Cnidaria | *Physalia*  *physalis* | S | Bardi & Marques 2007 | AF358065 |
| Cnidaria | *Podocoryna*  *carnea* | S | Boelsterli 1977 | JQ407393 |
| Cnidaria | *Porites*  *cylindrica* | S | Kerr et al. 2011 | KC816706 |
| Ctenophora | *Beroe*  *cucumis^τ^* | H | Sardet et al. 1990 | D15068 |
| Ctenophora | *Beroe*  *forskalii* | H | Oliveira & Migotto 2014 | AF293697 |
| Ctenophora | *Charistephane*  *fugiens* | H | Chun 1889 | AF293682 |
| Ctenophora | *Coeloplana*  *bannwarthii* | ? | Komai 1922 | AF293683 |
| Ctenophora | *Haeckelia*  *rubra* | H | Carré & Carré 1989 | AF293674 |
| Ctenophora | *Mertensia*  *ovum* | H | Jaspers et al. 2012 | AF293679 |
| Ctenophora | *Ocyropsis*  *maculata* | S | Harbison & Miller 1986 | AF293689 |
| Ctenophora | *Pleurobrachia*  *bachei* | H | Reeve & Walter 1978 | AF293677 |
| Ctenophora | *Pleurobrachia*  *pileus* | H | Fraser 1970 | AF293678 |
| Ctenophora | *Vallicula*  *multiformis* | H | Pianka 1974 | AF293684 |
| Ctenophora | *Velamen*  *parallelum* | H | Pianka 1974 | AF293693 |
| Deuterostomia | *Astrotoma*  *agassizii* | S | Maturana et al. 2016 | AB758520 |
| Deuterostomia | *Botryllus*  *schlosseri* | H | Rodriguez et al. 2016 | AB211066 |
| Deuterostomia | *Branchiostoma*  *floridae* | S | Stokes & Holland 1996 | M97571 |
| Deuterostomia | *Cephalodiscus*  *gracilis* | S | Dilly 2014 | AF236798 |
| Deuterostomia | *Ciona*  *intestinalis* | H | Caputi et al. 2015 | JN573244 |
| Deuterostomia | *Dumetocrinus antarcticus^ω^* | S | Messing & Dearborn 1990 | KC616753 |
| Deuterostomia | *Gallus*  *gallus* | S | Mignon-Grasteau et al. 1999 | AF173612 |
| Deuterostomia | *Homo*  *sapiens* | S | Collaer & Hines 1995 | X03205 |
| Deuterostomia | *Labidiaster annulatus* | ? |  | AY935548 |
| Deuterostomia | *Leptosynapta*  *clarki* | S | Sewell & Chia 1994 | DQ777083 |
| Deuterostomia | *Petromyzon marinus* | S | Buchinger et al. 2015 | M97575 |
| Deuterostomia | *Ptychodera bahamensis^τ^* | S | Tagawa et al. 1998 | AF236802 |
| Deuterostomia | *Saccoglossus mereschkowskii^τ^* | S | Burdon-Jones 1951 | KF683588 |
| Deuterostomia | *Schizocardium braziliense* | S | Franzen 2001 | KF683572 |
| Deuterostomia | *Strongylocentrotus purpuratus* | S | Levitan 2008 | L28055 |
| Ecdysozoa | *Daphnia*  *pulex* | S | Innes et al. 1986 | KJ775027 |
| Ecdysozoa | *Drosophila melanogaster* | S | Bateman 1948 | X70692 |
| Ecdysozoa | *Halicryptus spinulosus* | S | Jacob van der Land 1975 | AF342790 |
| Ecdysozoa | *Ixodes*  *acutitarsus^τ^* | S | Moorhouse 1966 | AF115364 |
| Ecdysozoa | *Peripatopsis capensis* | S | Manton 1938 | EU855526 |
| Ecdysozoa | *Priapulus*  *caudatus* | S | Wallace 2002 | AF025927 |
| Ecdysozoa | *Strigamia*  *maritima* | S | Lewis 1961 | AF173265 |
| Placozoa | *Trichoplax adhaerens* | ? | Schierwater 2005 | Z22783 |
| Porifera | *Acanthella*  *acuta* | ? | Riesgo et al. 2015 | 528272415 |
| Porifera | *Agelas*  *clathrodes* | ? | Riesgo et al. 2015 | 59939917 |
| Porifera | *Agelas*  *oroides* | S | Riesgo et al. 2015 | 528272442 |
| Porifera | *Amphimedon compressa* | H | Riesgo et al. 2015 | 528272435 |
| Porifera | *Amphimedon*  *erina* | H | Riesgo et al. 2015 | 528272436 |
| Porifera | *Aphrocallistes vastus* | H | Riesgo et al. 2015 | 528272414 |
| Porifera | *Aplysina*  *aerophoba* | S | Riesgo et al. 2015 | 528272421 |
| Porifera | *Aplysina cavernicola* | S | Riesgo et al. 2015 | 528272422 |
| Porifera | *Aplysina*  *fulva* | ? | Riesgo et al. 2015 | 528272450 |
| Porifera | *Asbestopluma occidentalis* | H | Riesgo et al. 2015 | 528272448 |
| Porifera | *Axinella*  *damicornis* | S | Riesgo et al. 2015 | 528272426 |
| Porifera | *Axinella*  *verrucosa* | S | Riesgo et al. 2015 | 302032324 |
| Porifera | *Calyx*  *podatypa* | ? | Riesgo et al. 2015 | 528272433 |
| Porifera | *Chalinula*  *molitba* | ? | Riesgo et al. 2015 | 528272430 |
| Porifera | *Chondrilla australiensis* | S | Riesgo et al. 2015 | 528272449 |
| Porifera | *Chondrilla*  *nucula* | S | Riesgo et al. 2015 | 187961570 |
| Porifera | *Chondrosia reniformis* | S | Riesgo et al. 2015 | 38327051 |
| Porifera | *Clathrina*  *clathrus* | H | Riesgo et al. 2015 | 528272451 |
| Porifera | *Cliona*  *viridis* | H | Riesgo et al. 2015 | 528272443 |
| Porifera | *Corticium candelabrum* | H | Riesgo et al. 2015 | 528272423 |
| Porifera | *Crambe*  *crambe* | H | Riesgo et al. 2015 | 150024063 |
| Porifera | *Dictyonella*  *incisa* | ? | Riesgo et al. 2015 | 38327055 |
| Porifera | *Dragmacidon lunaecharta* | ? | Riesgo et al. 2015 | 528272424 |
| Porifera | *Ephydatia*  *fluviatilis* | S | Riesgo et al. 2015 | 50403830 |
| Porifera | *Eunapius*  *fragilis* | ? | Riesgo et al. 2015 | 4585352 |
| Porifera | *Geodia*  *barretti* | S | Riesgo et al. 2015 | 528272447 |
| Porifera | *Halichondria okadai* | S | Riesgo et al. 2015 | 334351338 |
| Porifera | *Haliclona*  *elegans* | S | Riesgo et al. 2015 | 528272416 |
| Porifera | *Haliclona*  *oculata* | H | Riesgo et al. 2015 | AY734450 |
| Porifera | *Haliclona*  *sarai* | ? | Riesgo et al. 2015 | 528272441 |
| Porifera | *Haliclona*  *xena* | H | Riesgo et al. 2015 | 528272431 |
| Porifera | *Halisarca*  *dujardini* | H | Riesgo et al. 2015 | 187961575 |
| Porifera | *Heterochone*  *calyx* | ? | Riesgo et al. 2015 | 190350250 |
| Porifera | *Hexadella*  *pruvoti* | ? | Riesgo et al. 2015 | 528272445 |
| Porifera | *Hippospongia lachne* | S | Riesgo et al. 2015 | 187961576 |
| Porifera | *Hymeniacidon perlevis* | H | Riesgo et al. 2015 | 295650631 |
| Porifera | *Lissodendoryx colombiensis* | ? | Riesgo et al. 2015 | 528272440 |
| Porifera | *Lophocalyx profundum* | ? | Riesgo et al. 2015 | 190350236 |
| Porifera | *Microciona prolifera* | H | Riesgo et al. 2015 | 127692667 |
| Porifera | *Mycale*  *laxissima* | H | Riesgo et al. 2015 | 528272425 |
| Porifera | *Negombata magnifica* | H | Riesgo et al. 2015 | 528272444 |
| Porifera | *Nodastrella asconemaoida* | ? | Riesgo et al. 2015 | 190350231 |
| Porifera | *Oopsacas*  *minuta* | H | Riesgo et al. 2015 | 6694689 |
| Porifera | *Oscarella*  *lobularis* | H | Riesgo et al. 2015 | 316990467 |
| Porifera | *Petrosia*  *ficiformis* | S | Riesgo et al. 2015 | 528272432 |
| Porifera | *Placospongia intermedia* | ? | Riesgo et al. 2015 | 528272437 |
| Porifera | *Rhopaloeides odorabile* | S | Riesgo et al. 2015 | 528272439 |
| Porifera | *Scopalina*  *ruetzleri* | H | Riesgo et al. 2015 | 528272418 |
| Porifera | *Siphonochalina siphonella* | H | Riesgo et al. 2015 | 528272419 |
| Porifera | *Spirastrella cunctatrix* | S | Riesgo et al. 2015 | 528272417 |
| Porifera | *Spongia*  *officinalis* | S | Riesgo et al. 2015 | 38327063 |
| Porifera | *Spongilla*  *lacustris* | S | Riesgo et al. 2015 | 77379381 |
| Porifera | *Suberites domuncula* | S | Riesgo et al. 2015 | 55580998 |
| Porifera | *Suberites*  *ficus* | S | Riesgo et al. 2015 | 4093179 |
| Porifera | *Sycon*  *coactum* | H | Riesgo et al. 2015 | 528272446 |
| Porifera | *Tedania*  *ignis* | ? | Riesgo et al. 2015 | 53771880 |
| Porifera | *Tethya*  *aurantium* | S | Riesgo et al. 2015 | 528272428 |
| Porifera | *Tethya*  *citrina* | S | Riesgo et al. 2015 | 528272429 |
| Porifera | *Tethya*  *sp. 1* | ? | Riesgo et al. 2015 | 528272420 |
| Porifera | *Tethya*  *sp. 2* | ? | Riesgo et al. 2015 | 528272438 |
| Porifera | *Thenea*  *muricata* | S | Riesgo et al. 2015 | 528272427 |
| Porifera | *Xestospongia*  *muta* | S | Riesgo et al. 2015 | 528272434 |
| Spiralia | *Adineta*  *ricciae* | A | Welch & meselson 2000 | KM043251 |
| Spiralia | *Adineta*  *vaga* | A | Welch & meselson 2000 | KM043254 |
| Spiralia | *Barentsia*  *gracilis* | S | Mariscal 1965 | FJ196109 |
| Spiralia | *Brachionus calyciflorus* | S | Radix et al. 2002 | DQ297692 |
| Spiralia | *Capitella*  *teleta* | S | Blake et al. 2009 | JF509728 |
| Spiralia | *Cephalothrix hongkongiensis* | S | Li et al. 2014 | JF293057 |
| Spiralia | *Crassostrea*  *gigas* | S | Castanos et al. 2009 | AB064942 |
| Spiralia | *Dugesia mediterranea* | H | Chong et al. 2013 | U31084 |
| Spiralia | *Helobdella nununununojensis* | H | Weisblat & Kuo 2014 | AY962426\| |
| Spiralia | *Hemithiris psittaceae* | S | James et al. 1992 | U08322 |
| Spiralia | *Lepidodermella squamata* | A | Ulbrich 1977 | JN185472 |
| Spiralia | *Leptochiton*  *rugatus* | S | Eernisse 2007 | HQ907769 |
| Spiralia | *Lineus*  *bilineatus* | S | Fish & Fish 1989 | DQ279932 |
| Spiralia | *Lottia*  *gigantea* | S | Kido & Murray 2003 | AB282762 |
| Spiralia | *Loxosoma pectinaricola* | S | Mariscal 1965 | GU125748 |
| Spiralia | *Macrodasys*  *sp.* | H | Ruppert 1978 | JF357654 |
| Spiralia | *Macrostomum lignano* | H | Janicke & Sharer 2010 | FJ715306 |
| Spiralia | *Megadasys*  *sp.* | H | Guidi et al. 2014 | JF357656 |
| Spiralia | *Membranipora membranacea* | ? | Harvell et al. 1990 - G  Temkin 1994 - H | FJ196121 |
| Spiralia | *Novocrania anomala* | S | Nielsen 1991 | AY842018 |
| Spiralia | *Phoronis psammophila* | S | Emig 1982 | U36271 |
| Spiralia | *Pomatoceros lamarckii* | S | Cotter et al. 2003 | DQ140404\| |
| Spiralia | *Prostheceraeus vittatus* | H | Norena et al. 2014 | AJ312272 |
| Spiralia | *Schistosoma mansoni* | S | Popiel et al. 1984 | U65657 |
| Spiralia | *Taenia*  *pisiformis^τ^* | H | Smith et al. 1972 | JQ609339 |
| Spiralia | *Terebratalia transversa* | S | James et al. 1992 | AF025945 |
| Xenacoelomorpha | *Ascoparia*  *sp.* | H | Sterrer 1998 | FR837678 |
| Xenacoelomorpha | *Childia submaculatum^τ^* | H | Nilsson et al. 2011 | AY297953 |
| Xenacoelomorpha | *Convolutriloba macropyga* | H | Shannon & Achatz 2007 | EU710912 |
| Xenacoelomorpha | *Diopisthoporus longitubus* | H | Jondelius et al. 2011 | FR837692 |
| Xenacoelomorpha | *Diopisthoporus psammophilus* | H | Jondelius et al. 2011 | FR837694 |
| Xenacoelomorpha | *Eumecynostomum altitudi^ω^* | H | Dorjes & Karling 1975 | AY297955 |
| Xenacoelomorpha | *Hofstenia*  *miamia* | H | Corrêa 1960 | AM701807 |
| Xenacoelomorpha | *Isodiametra pulchra* | H | Boone et al. 2011 | FR837710 |
| Xenacoelomorpha | *Meara*  *stichopi* | H | Sterrer 1998 | AF119085 |
| Xenacoelomorpha | *Nemertoderma westbladi* | H | Sterrer 1998 | AF327726 |
| Xenacoelomorpha | *Sterreria*  *sp.* | H | Meyer-Wachsmuth et al. 2014 | KM062555\| |
| Xenacoelomorpha | *Xenoturbella*  *bocki* | H | Westblad 1949 | AY291292 |

**Literature Cited**

Bardi J, Marques AC. 2007. Taxonomic redescription of the Portuguese man-of-war, *Physalia physalis* (Cnidaria, Hydrozoa, Siphonophorae, Cystonectae) from Brazil. Iheringia. Sér Zool. 97(4):425-433.

Bateman A. 1948. lntra-sexual selection in *Drosophila*. Heredity (Edinb.) 2(3):349-68.

Blake JA, Grassle JP, Eckelbarger KJ. 2009. Capitella teleta, a new species designation for the opportunistic and experimental *Capitella sp. I*, with a review of the literature for confirmed records. Zoosymposia 2(1):25-53.

Boelsterli U. 1977. An electron microscopic study of early developmental stages, myogenesis, oogenesis and cnidogenesis in the anthomedusa, *Podocoryne carnea* M. Sars. J Morphol. 154(2):259-289.

Boone M, Bert W, Claeys M, Houthoofd W, Artois T. 2011. Spermatogenesis and the structure of the testes in Nemertodermatida. Zoomorphology 130(4):273-282.

Buchinger TJ, Siefkes MJ, Zielinski BS, Brant CO, Li W. 2015. Chemical cues and pheromones in the sea lamprey (*Petromyzon marinus*). Front Zool. 12(1):1.

Burdon-Jones C. 1951. Observations on the spawning behaviour of *Saccoglossus horsti* Brambell & Goodhart, and of other Enteropneusta. J Mar Biol Assoc UK. 29(03):625-638.

Caputi L, Crocetta F, Toscano F, Sordino P, Cirino P. 2015. Long‐term demographic and reproductive trends in *Ciona intestinalis sp. A*. Mar Ecol. 36(1):118-128.

Carré D, Carré C. 1989. Acquisition de cnidocystes et différenciation de pseudocolloblastes chez les larves et les adultes de deux cténophores du genre Haeckelia Carus, 1863. Can J Zool. 67(9):2169-2179.

Carré D, Carré C. 2000. Origin of germ cells, sex determination, and sex inversion in medusae of the genus Clytia (Hydrozoa, leptomedusae): the influence of temperature. J Exp Zool. 287(3):233-242.

Castaños C, Pascual M, Camacho AP. 2009. Reproductive biology of the nonnative oyster, *Crassostrea gigas* (Thunberg, 1793), as a key factor for its successful spread along the rocky shores of northern Patagonia, Argentina. J Shellfish Res. 28(4):837-847.

Chong T, Collins III JJ, Brubacher JL, Zarkower D, Newmark PA. 2013. A sex-specific transcription factor controls male identity in a simultaneous hermaphrodite. Nat Comm. 4:1814.

Chun K. 1898. Die Ctenophoren der Plankton-expedition. Lipsius & Tischer.

Collaer ML, Hines M. 1995. Human behavioral sex differences: a role for gonadal hormones during early development? Psychol Bull. 118(1):55.

Cordes E, Nybakken J, VanDykhuizen G. 2001. Reproduction and growth of *Anthomastus ritteri* (Octocorallia: Alcyonacea) from Monterey Bay, California, USA. Mar Biol. 138(3):491-501.

Corrêa DD. 1960. Two new marine Turbellaria from Florida. Bull Mar Sci. 10(2):208-216.

Cotter E, O'Riordan R, Myers A. 2003. A histological study of reproduction in the serpulids *Pomatoceros triqueter* and *Pomatoceros lamarckii* (Annelida: Polychaeta). Mar Biol. 142(5):905-914.

Dilly PN. 2014. Cephalodiscus reproductive biology (Pterobranchia, Hemichordata). Acta Zool. 95(1):111-124.

Dörjes J, Karling TG. 1975. Species of Turbellaria Acoela in the Swedish Museum of Natural History, with remarks on their anatomy, taxonomy and distribution. Zool. Scr. 4(1):175-189.

Dunn CW, Pugh PR, Haddock SH. 2005. Molecular phylogenetics of the siphonophora (Cnidaria), with implications for the evolution of functional specialization. Syst Biol. 54(6):916-935.

Eckelbarger KJ, Larson RJ. 1993. Ultrastructural study of the ovary of the sessile scyphozoan, *Haliclystus octoradiatus* (Cnidaria: Stauromedusae). J Morphol. 218(2):225-236.

Eernisse D. 2007. Chitons. Encyclopedia of tidepools and rocky shores:127-133.

Emig C. 1982. The biology of Phoronida. Adv Mar Biol. 19:1-89.

Fish J, Fish S. 1989. Nemertea. A Student’s Guide to the Seashore. Springer. p. 129-133.

Franzen Å. 2001. Sperm ultrastructure in the enteropneust *Schizocardium sp* (Hemichordata, Enteropneusta) and possible phylogenetic implications. Invertebr Reprod Dev.39(1):37-43.

Fraser J. 1970. The ecology of the ctenophore *Pleurobrachia pileus* in Scottish waters. J Conseil. 33(2):149-168.

Grawunder D, Hambleton EA, Bucher M, Wolfowicz I, Bechtoldt N, Guse A. 2015. Induction of Gametogenesis in the Cnidarian Endosymbiosis Model Aiptasia sp. Sci Rep. 5.

Grosberg RK, Levitan DR, Cameron BB. 1996. Evolutionary genetics of allorecognition in the colonial hydroid *Hydractinia symbiolongicarpus*. Evolution. 2221-2240.

Guidi L, Todaro MA, Ferraguti M, Balsamo M. 2014. Reproductive system and spermatozoa ultrastructure support the phylogenetic proximity of Megadasys and Crasiella (Gastrotricha, Macrodasyida). Contrib Zool. 83(2).

Hand C, Uhlinger KR. 1992. The culture, sexual and asexual reproduction, and growth of the sea anemone *Nematostella vectensis*. Biol Bull. 182(2):169-176.

Harbison G, Miller R. 1986. Not all ctenophores are hermaphrodites. Studies on the systematics, distribution, sexuality and development of two species of *Ocyropsis*. Mar Biol. 90(3):413-424.

Harvell CD, Caswell H, Simpson P. 1990. Density effects in a colonial monoculture: experimental studies with a marine bryozoan (*Membranipora membranacea L.).* Oecologia 82(2):227-237.

Hinsch GW, Moore JA. 1992. The structure of the reproductive mesenteries of the sea anemone *Ceriantheopsis americanus*. Invertebr Reprod Dev. 21(1):25-32.

Hwang S-J, Song J-I. 2007. Reproductive biology and larval development of the temperate soft coral *Dendronephthya gigantea* (Alcyonacea: Nephtheidae). Mar Biol. 152(2):273-284.

Hwang SJ, Song JI. 2009. Sexual reproduction of soft coral, Scleronephthya gracillimum,(Alcyonacea: Nephtheidae) based on long-term collection from Jejudo Island, Korea. Galaxea, J Coral Reef Stud. 11(2):155-167.

Innes DJ, Schwartz SS, Hebert PDN. 1986. Genotypic diversity and variation in mode of reproduction among populations in the *Daphnia pulex* group. Heredity (Edinb.) 57:345-355.

James M, Ansell A, Collins M, Curry G, Peck L, Rhodes M. 1992. Biology of living brachiopods. Adv Mar Biol. 28:175-387.

Janicke T, Schärer L. 2010. Sperm competition affects sex allocation but not sperm morphology in a flatworm. Behav Ecol Sociobiol. 64(9):1367-1375.

Jaspers C, Haraldsson M, Bolte S, Reusch TB, Thygesen UH, Kiørboe T. 2012. Ctenophore population recruits entirely through larval reproduction in the central Baltic Sea. Biol Lett. rsbl20120163.

Jondelius U, Wallberg A, Hooge M, Raikova OI. 2011. How the worm got its pharynx: phylogeny, classification and Bayesian assessment of character evolution in Acoela. Syst Biol. syr073.

Kaplan SW. 1983. Intrasexual aggression in *Metridium senile*. Biol. Bull. 165(2):416-418.

Kerr AM, Baird AH, Hughes TP. 2011. Correlated evolution of sex and reproductive mode in corals (Anthozoa: Scleractinia). P Roy Soc Lond B Bio. 278(1702):75-81.

Kido JS, Murray SN. 2003. Variation in owl limpet *Lottia gigantea* population structures, growth rates, and gonadal production on southern California rocky shores. Mar Ecol Prog Ser. 257:111-124.

Kingsford MJ, Mooney CJ. 2014. The ecology of box jellyfishes (Cubozoa). Jellyfish Blooms. Springer. p. 267-302.

Komai T. 1922. Studies on two aberrant ctenophores: Coeloplana and Gastrodes. The author.

Levitan DR. 2008. Gamete traits influence the variance in reproductive success, the intensity of sexual selection, and the outcome of sexual conflict among congeneric sea urchins. Evolution 62(6):1305-1316.

Levitan DR, Fukami H, Jara J, Kline D, McGovern TM, McGhee KE, Swanson CA, Knowlton N. 2004. Mechanisms of reproductive isolation among sympatric broadcast‐spawning corals of the *Montastraea annularis* species complex. Evolution 58(2):308-323.

Lewis JGE. The life history and ecology of the littoral centipede *Strigamia* (= *Scolioplanes*) *maritima* (Leach). P Zool Soc Lond. 1961: Wiley Online Library. p. 221-248.

Li Y, Song X, Zhang S, Zhu L, Sun S. 2014. Effect of temperature and photoperiod on maturation of *Cephalothrix hongkongiensis* (Nemertea: Palaeonemertea). Chin J Oceanol Limn. 32:764-772.

Lucas CH. 2001. Reproduction and life history strategies of the common jellyfish, *Aurelia aurita*, in relation to its ambient environment. Hydrobiologia 451(1-3):229-246.

Lucas CH, Reed AJ. 2010. Gonad morphology and gametogenesis in the deep-sea jellyfish *Atolla wyvillei* and *Periphylla periphylla* (Scyphozoa: Coronatae) collected from Cape Hatteras and the Gulf of Mexico. J Mar Biol. Assoc. UK 90(06):1095-1104.

Manton S. 1938. Studies on the Onychophora. IV. The passage of spermatozoa into the ovary in Peripatopsis and the early development of the ova. Philos T Roy Soc B. :421-441.

Mariscal RN. 1965. The adult and larval morphology and life history of the entoproct *Barentsia gracilis* (M. Sars, 1835). J Morphol. 116(3):311-338.

Maturana CS, Gérard K, Díaz A, David B, Féral J-P, Poulin E. 2016. Mating system and evidence of multiple paternity in the Antarctic brooding sea urchin *Abatus agassizii.* Polar Biol.:1-11.

Messing CG, Dearborn JH. 1990. Marine Flora and Fauna of the Northeastern United States Echinodermata: Crinoidea. NOAA Technical Report:1.

Meyer-Wachsmuth I, Galletti MC, Jondelius U. 2014. Hyper-cryptic marine meiofauna: species complexes in Nemertodermatida. PLoS One 9(9):e107688.

Mignon-Grasteau S. 1999. Genetic parameters of growth curve parameters in male and female chickens. Br Poult Sci. 40(1):44-51.

Moorhouse DE. 1966. Observations on copulation in *Ixodes holocyclus* Neumann and the feeding of the male. J Med Entomol. 3(2):168-171.

Nawrocki AM. 2012. Phylogenetics of Aplanulata (Cnidaria: Hydrozoa) and the evolution and development of *Ectopleura larynx*. University of Kansas.

Nielsen C. 1991. The development of the brachiopod *Crania* (*Neocrania*) *anomala* (OF Müller) and its phylogenetic significance. Acta Zool. 72(1):7-28.

Nilsson KS, Wallberg A, Jondelius U. 2011. New species of Acoela from the Mediterranean, the Red Sea, and the South Pacific. Zootaxa 2867(1):1-31.

Noreña C, Marquina D, Perez J, Almon B. 2014. First records of Cotylea (Polycladida, Platyhelminthes) for the Atlantic coast of the Iberian Peninsula. ZooKeys(404):1.

Oliveira OMPd, Migotto AE. 2014. First occurrence of *Beroe forskalii* (Ctenophora) in South American Atlantic coastal waters, with notes on the use of macrociliary patterns for beroid identification. Zootaxa 3779(4):470-476.

Pianka HD. 1974. Ctenophora. In: Giese A, Pearse J, editors. Reproduction of marine invertebrates Vol. 1. New York, NY: Academic Press, Inc. p. 201-265.

Popiel I, Cioli D, Erasmus DA. 1984. The morphology and reproductive status of female *Schistosoma mansoni* following separation from male worms. Int J Parasitol. 14(2):183-190.

Prada C, Hellberg ME. 2013. Long prereproductive selection and divergence by depth in a Caribbean candelabrum coral. P Natl Acad Sci. 110(10):3961-3966.

Radix P, Severin G, Schramm K-W, Kettrup A. 2002. Reproduction disturbances of *Brachionus calyciflorus* (rotifer) for the screening of environmental endocrine disrupters. Chemosphere 47(10):1097-1101.

Reeve M, Walter M. 1979. Nutritional ecology of ctenophores—a review of recent research. Adv Mar Biol. 15:249-287.

Riesgo A, Novo M, Sharma PP, Peterson M, Maldonado M, Giribet G. 2014. Inferring the ancestral sexuality and reproductive condition in sponges (Porifera). Zool Scr. 43(1):101-117.

Rodriguez D, Kassmer SH, De Tomaso AW. 2016. Gonad development and hermaphroditism in the ascidian *Botryllus schlosseri*. Mol Reprod Dev.

Ruppert EE. 1978. The reproductive system of gastrotrichs. Zoomorphologie 89(3):207-228.

Sardet C, Carré D, Rouvière C. 1990. Reproduction and development in ctenophores. Experimental Embryology in Aquatic Plants and Animals. Springer. p. 83-94.

Schierwater B. 2005. My favorite animal, *Trichoplax adhaerens*. Bioessays 27(12):1294-1302.

Sewell M, Chia F-S. 1994. Reproduction of the intraovarian brooding apodid *Leptosynapta clarki* (Echinodermata: Holothuroidea) in British Columbia. Mar Biol. 121(2):285-300.

Shannon T, Achatz JG. 2007. Convolutriloba macropyga sp nov., an uncommonly fecund acoel (Acoelomorpha) discovered in tropical aquaria. Zootaxa 1525(1):1-17.

Shick JM. 2012. A functional biology of sea anemones. Springer Science & Business Media.

Smith JK, Esch GW, Kuhn RE. 1972. Growth and development of larval *Taenia crassiceps* (Cestoda)—I.: Aneuploidy in the anomalous orf strain. Int J Parasitol. 2(2):261-262.

Sterrer W. 1998. New and known Nemertodermatida (Platyhelminthes-Acoelomorpha)-a revision. Belg J Zool. 128:55-92.

Stokes MD, Holland ND. 1996. Reproduction of the Florida lancelet (*Branchiostoma floridae):* spawning patterns and fluctuations in gonad indexes and nutritional reserves. Invertebr Biol.:349-359.

Sugiyama T, Fujisawa T. 1977. Genetic analysis of developmental mechanisms in hydra I. Sexual reproduction of *Hydra magnipapillata* and isolation of mutants. Dev Growth Differ. 19(3):187-200.

Tagawa K, Nishino A, Humphreys T, Satoh N. 1998. The spawning and early development of the Hawaiian acorn worm (Hemichordate), *Ptychodera flava*. Zoolog Sci. 15(1):85-91.

Temkin M. 1994. Gamete spawning and fertilization in the gymnolaemate bryozoan *Membranipora membranacea*. Biol Bull. 187(2):143-155.

Ulbrich RW. 1977. The Microscopic Anatomy of Lepidodermella squamata (Dujardin, 1841). [Loyola eCommons]: Loyola University Chicago.

Van Der Land J. 1974. Priapulida. In: Giese A, Pearse J, editors. Reproduction of Marine Invertebrates Vol. 2. New York, NY: Academic Press, Inc. p. 55-65.

Wagner D, Waller R, Montgomery A, Kelley C, Toonen R. 2012. Sexual reproduction of the Hawaiian black coral *Antipathes griggi* (Cnidaria: Antipatharia). Coral reefs 31(3):795-806.

Wallace RL. Priapulida. eLS.

Weisblat DA, Kuo D-H. 2014. Developmental biology of the leech *Helobdella*. The Int J Dev Biol. 58:429.

Welch DBM, Meselson M. 2000. Evidence for the evolution of bdelloid rotifers without sexual reproduction or genetic exchange. Science 288(5469):1211-1215.

Westblad E. 1949. *Xenoturbella bocki* ng, n. sp., a peculiar, primitive turbellarian type. Arkiv för zoologi. 1:11-29.
