# Supplementary figures and images for: A reconstruction of sexual modes throughout animal evolution
Source: BMC Evol Biol. 2017 Dec 6;17:242. doi: 10.1186/s12862-017-1071-3 (PMC5717846; doi:10.1186/s12862-017-1071-3)

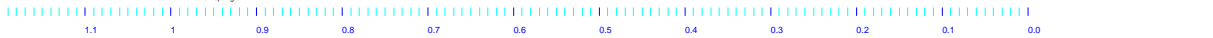

Supplement: Supplementary file 2 — Composite “ctenophore-sister” tree showing branch lengths generated from the use of one gene (18S). Figure generated using FigTree (http://tree.bio.ed.ac.uk/software/figtree/). (PDF 13 kb) [file 12862_2017_1071_MOESM2_ESM.pdf]

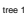

Supplement: Supplementary file 3 — Composite “sponge-sister” tree showing branch lengths generated from the use of one gene (18S). Figure generated using FigTree (http://tree.bio.ed.ac.uk/software/figtree/). (PDF 9 kb) [file 12862_2017_1071_MOESM3_ESM.pdf]

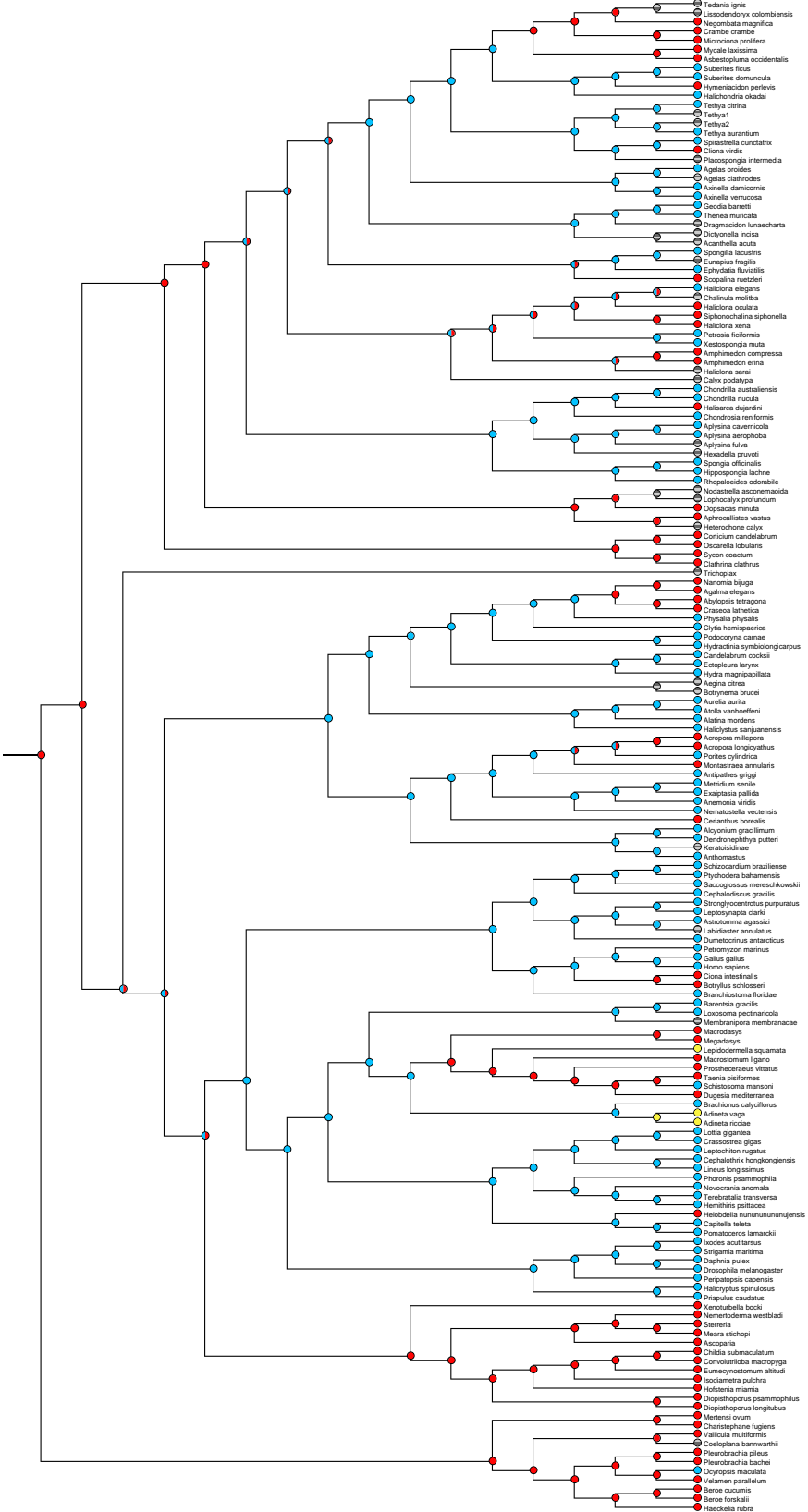

Supplement: Supplementary file 4 — Full cladogram of maximum-parsimony ancestral-state reconstruction of sexual mode for the “ctenophore-sister” topology using Mesquite. The color blue represents gonochorism, red represents hermaphroditism, yellow represents asexuality, and grey represents an unknown sexual mode. (PDF 78 kb) [file 12862_2017_1071_MOESM4_ESM.pdf]

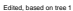

Supplement: Supplementary file 5 — Full cladogram of maximum-parsimony ancestral-state reconstruction of sexual mode for the “sponge-sister” topology using Mesquite. The color blue represents gonochorism, red represents hermaphroditism, yellow represents asexuality, and grey represents an unknown sexual mode. (PDF 78 kb) [file 12862_2017_1071_MOESM5_ESM.pdf]

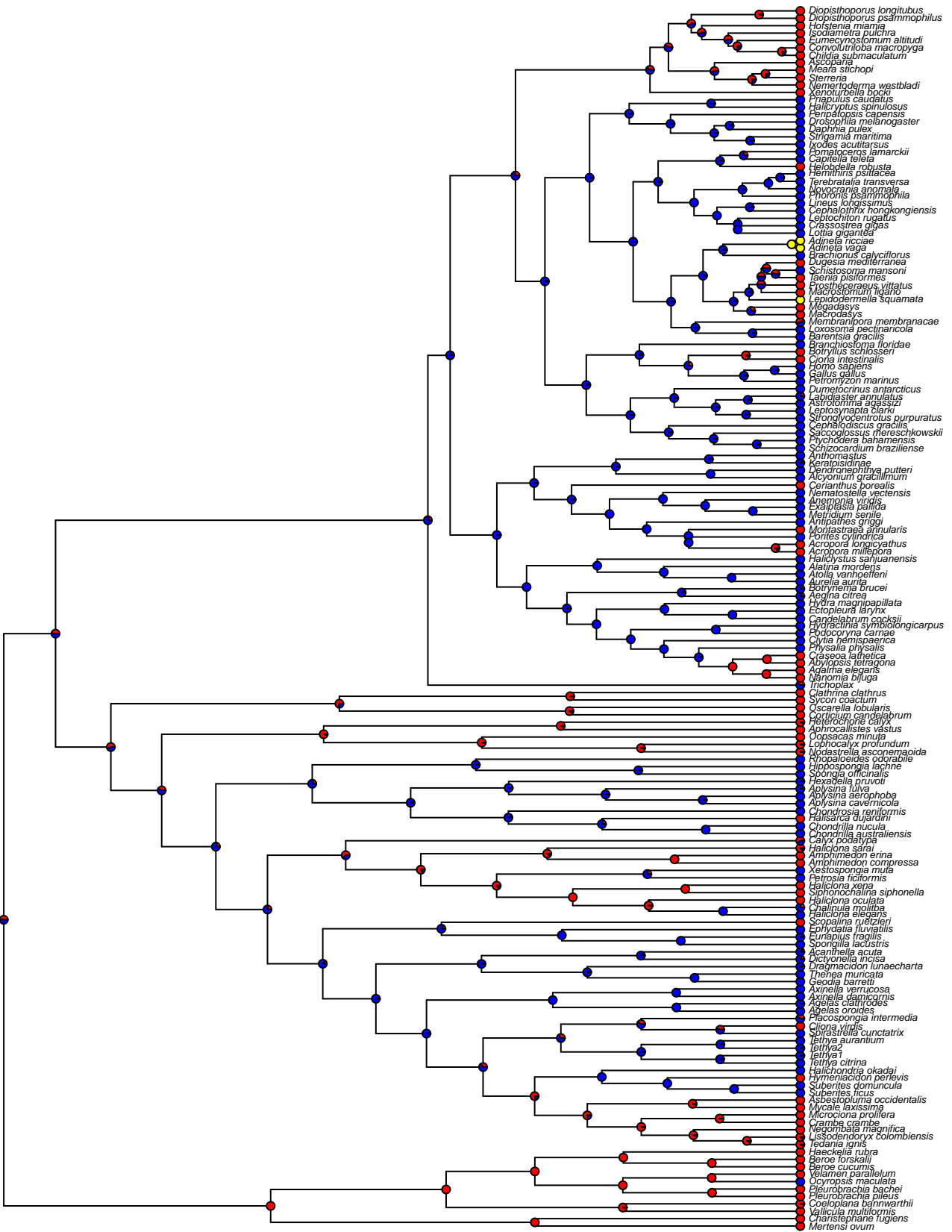

Supplement: Supplementary file 6 — Ancestral reconstruction of sexual mode on the “ctenophore-sister” topology from stochastic character mapping. The color blue represents gonochorism, red represents hermaphroditism, and yellow represents asexuality. We used a symmetrical rates model in the make_simmap function from the phytools package. Tree was converted to ultrametric using the ‘chronopl’ command in the Ape package. R script used to generate this PDF is available here: https://github.com/josephryan/2017b_Sasson_and_Ryan. (PDF 139 kb) [file 12862_2017_1071_MOESM6_ESM.pdf]

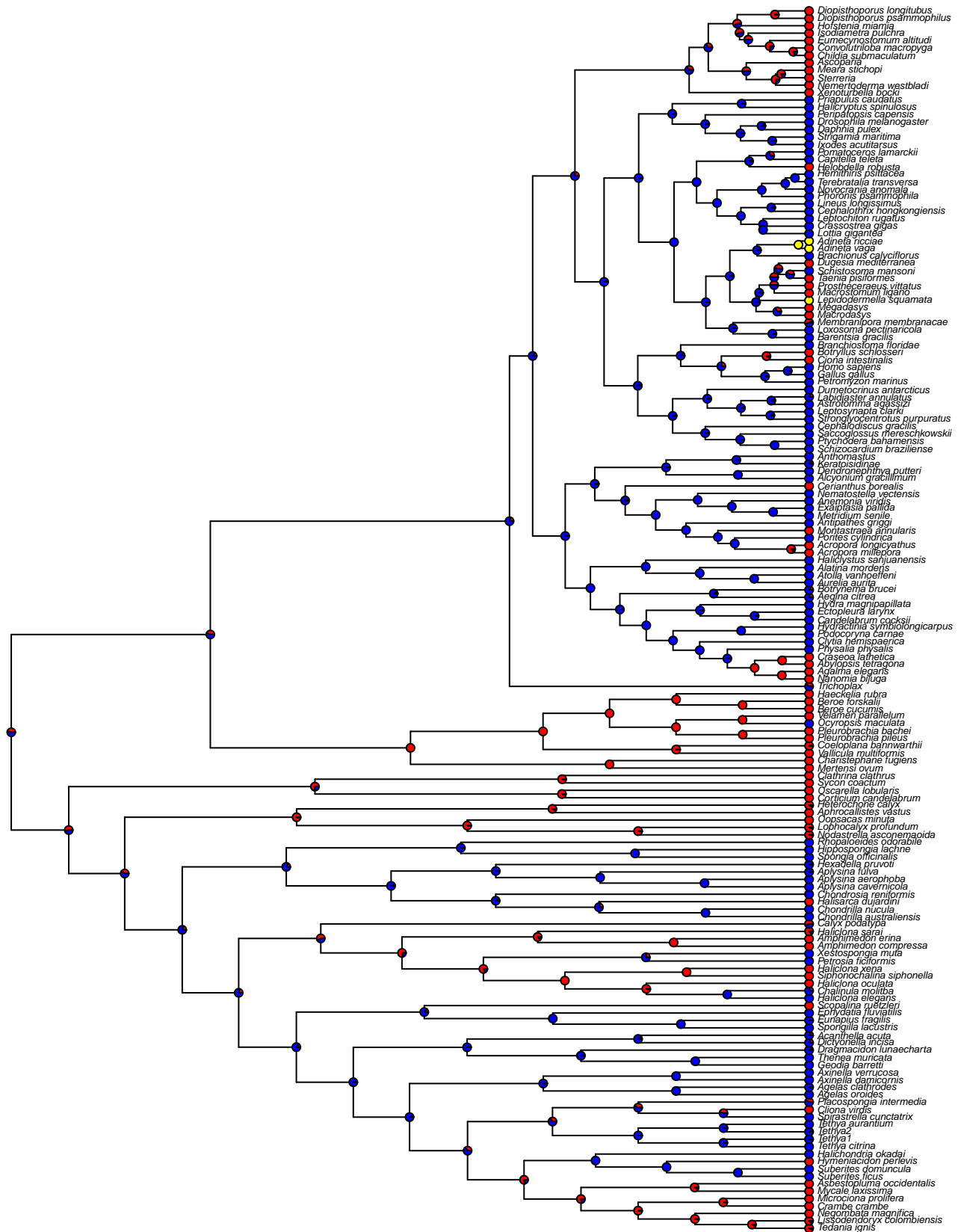

Supplement: Supplementary file 7 — Ancestral reconstruction of sexual mode on the “sponge-sister” topology from stochastic character mapping. The color blue represents gonochorism, red represents hermaphroditism, and yellow represents asexuality. We used a symmetrical rates model in the make_simmap function from the phytools package. Tree was converted to ultrametric using the ‘chronopl’ command in the Ape package. R script used to generate this PDF is available here: https://github.com/josephryan/2017b_Sasson_and_Ryan. (PDF 135 kb) [file 12862_2017_1071_MOESM7_ESM.pdf]

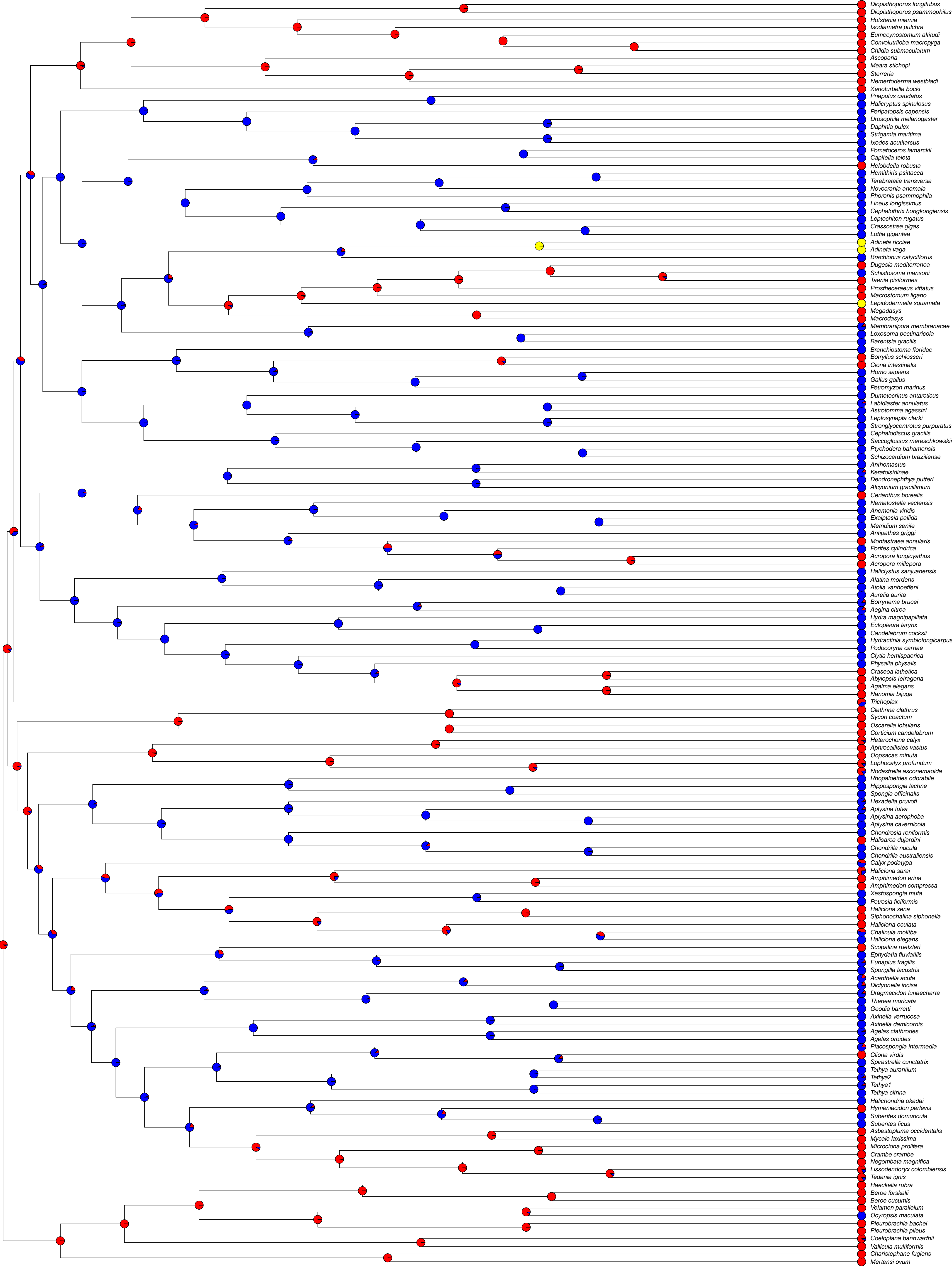

Supplement: Supplementary file 9 — Effect of branch lengths shown through ancestral state reconstruction of sexual mode on the “ctenophore-sister” topology without branch lengths. This analysis is the same as Additional file 6: Figure S5 except branch lengths were equal in this analysis. R script used to generate this PDF is available here: https://github.com/josephryan/2017b_Sasson_and_Ryan. (PDF 187 kb) [file 12862_2017_1071_MOESM9_ESM.pdf]

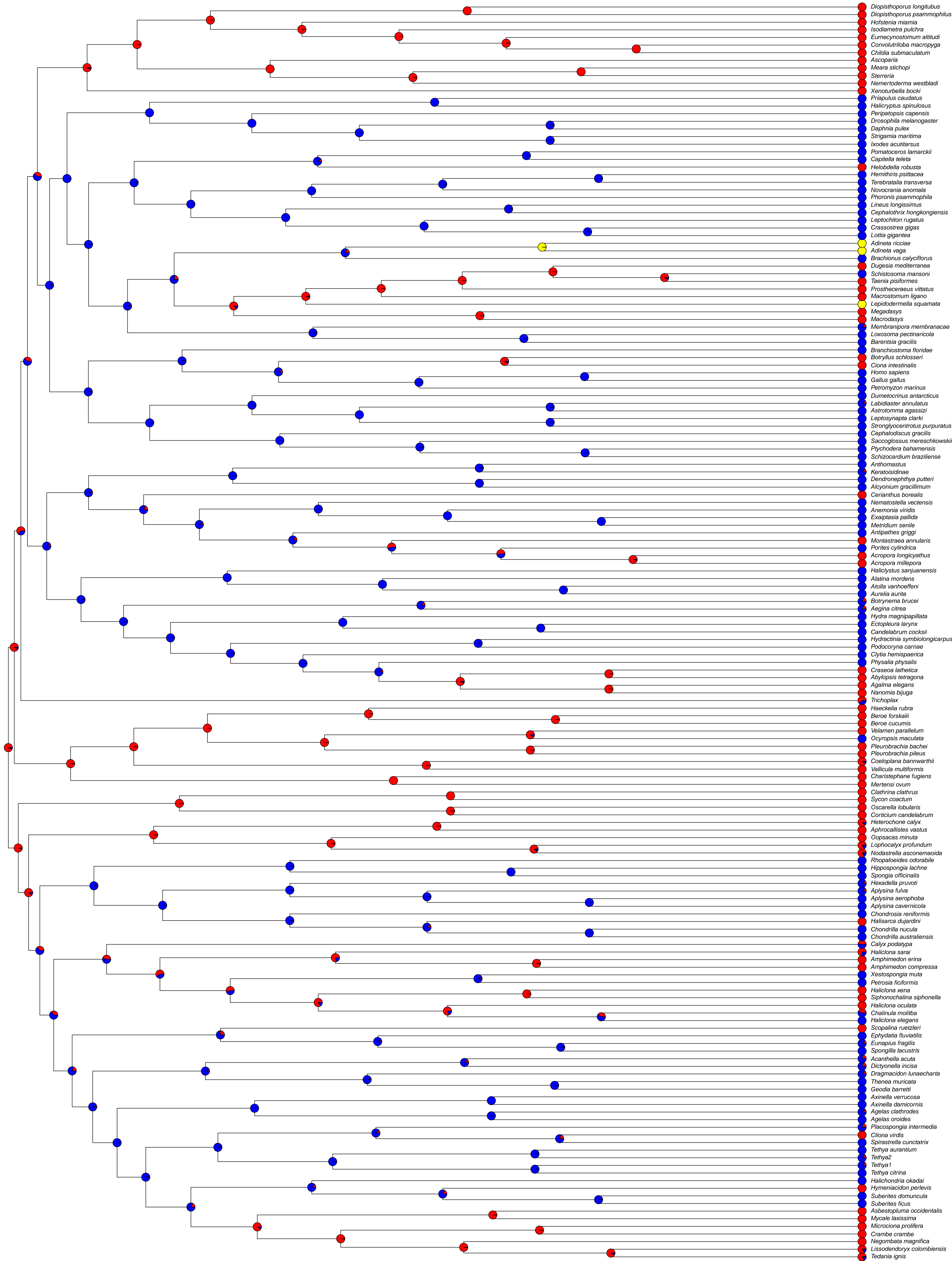

Supplement: Supplementary file 10 — Effect of branch lengths shown through ancestral state reconstruction of sexual mode on the “sponge-sister” topology with equal branch lengths. This analysis same as Additional file 7: Figure S6 except branch lengths were equalized in this the analysis. R script used to generate this PDF is available here: https://github.com/josephryan/2017b_Sasson_and_Ryan. (PDF 190 kb) [file 12862_2017_1071_MOESM10_ESM.pdf]

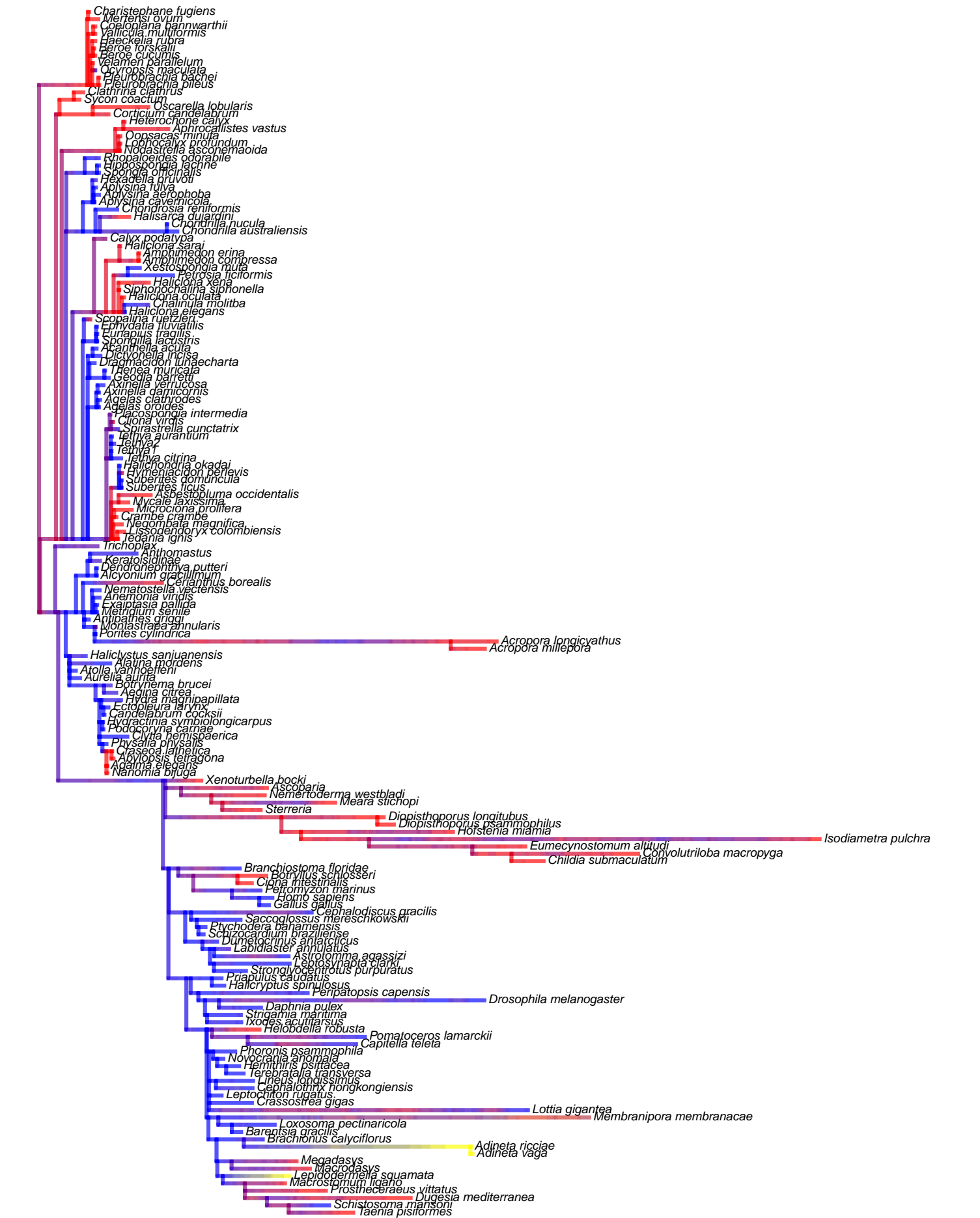

1.2 1.0 0.8 0.6 0.4 0.2 0.0 -0.2

Supplement: Supplementary file 11 — Transitions across the stochastic character mapping ancestral state reconstruction of the “ctenophore-sister” topology averaged over 1000 simulations. Blue represents separate sexes, red hermaphroditism, and yellow asexuality. Gradations between those colors indicate areas where transitions were simulated to have occurred. R script used to generate this PDF is available here: https://github.com/josephryan/2017b_Sasson_and_Ryan. (PDF 64 kb) [file 12862_2017_1071_MOESM11_ESM.pdf]

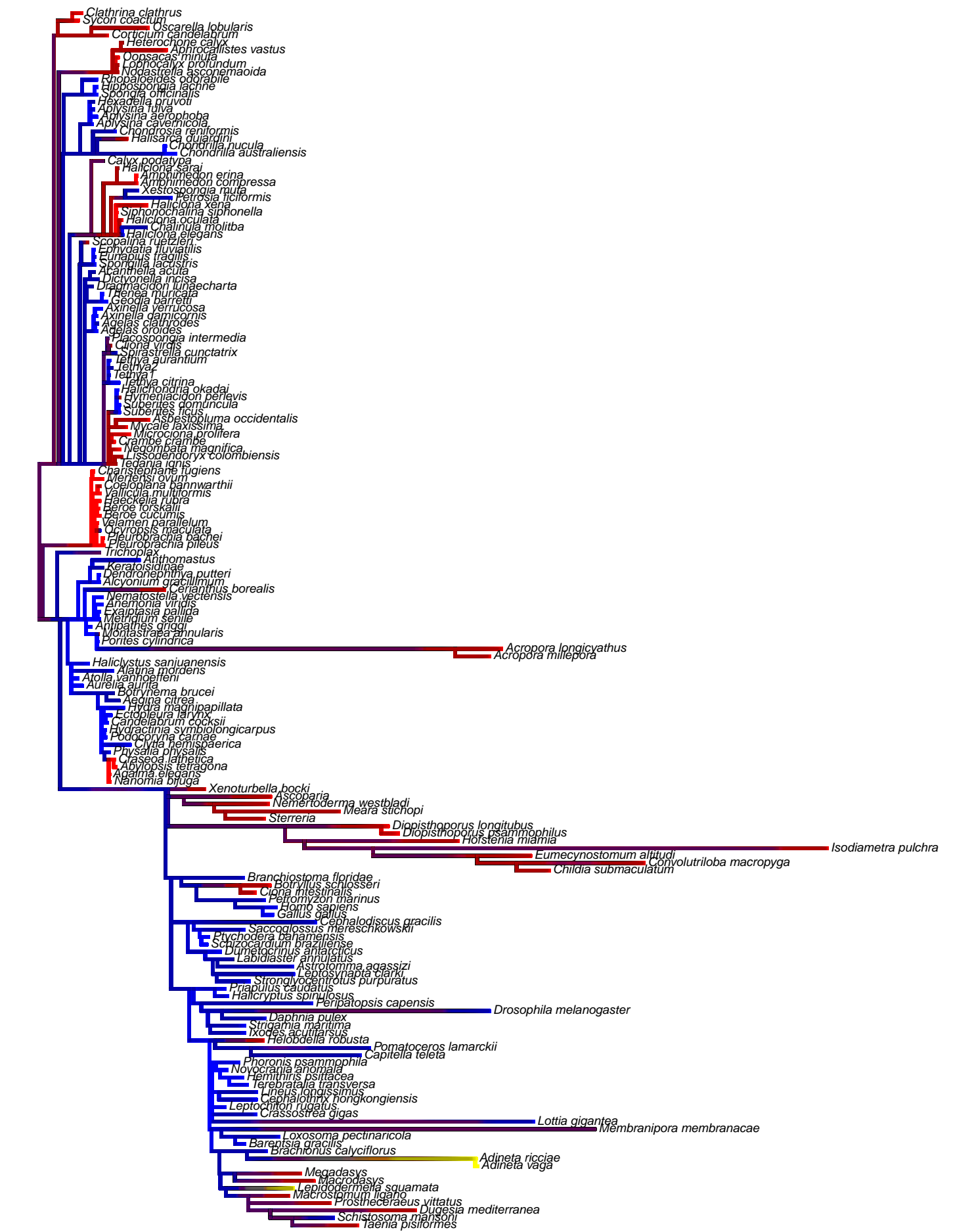

1.2 1.0 0.8 0.6 0.4 0.2 0.0 -0.2

Supplement: Supplementary file 12 — Transitions across the stochastic character mapping ancestral state reconstruction of the “sponge-sister” topology averaged over 1000 simulations. Blue represents separate sexes, red hermaphroditism, and yellow asexuality. Gradations between those colors indicate areas where transitions were simulated to have occurred. R script used to generate this PDF is available here: https://github.com/josephryan/2017b_Sasson_and_Ryan. (PDF 4 kb) [file 12862_2017_1071_MOESM12_ESM.pdf]

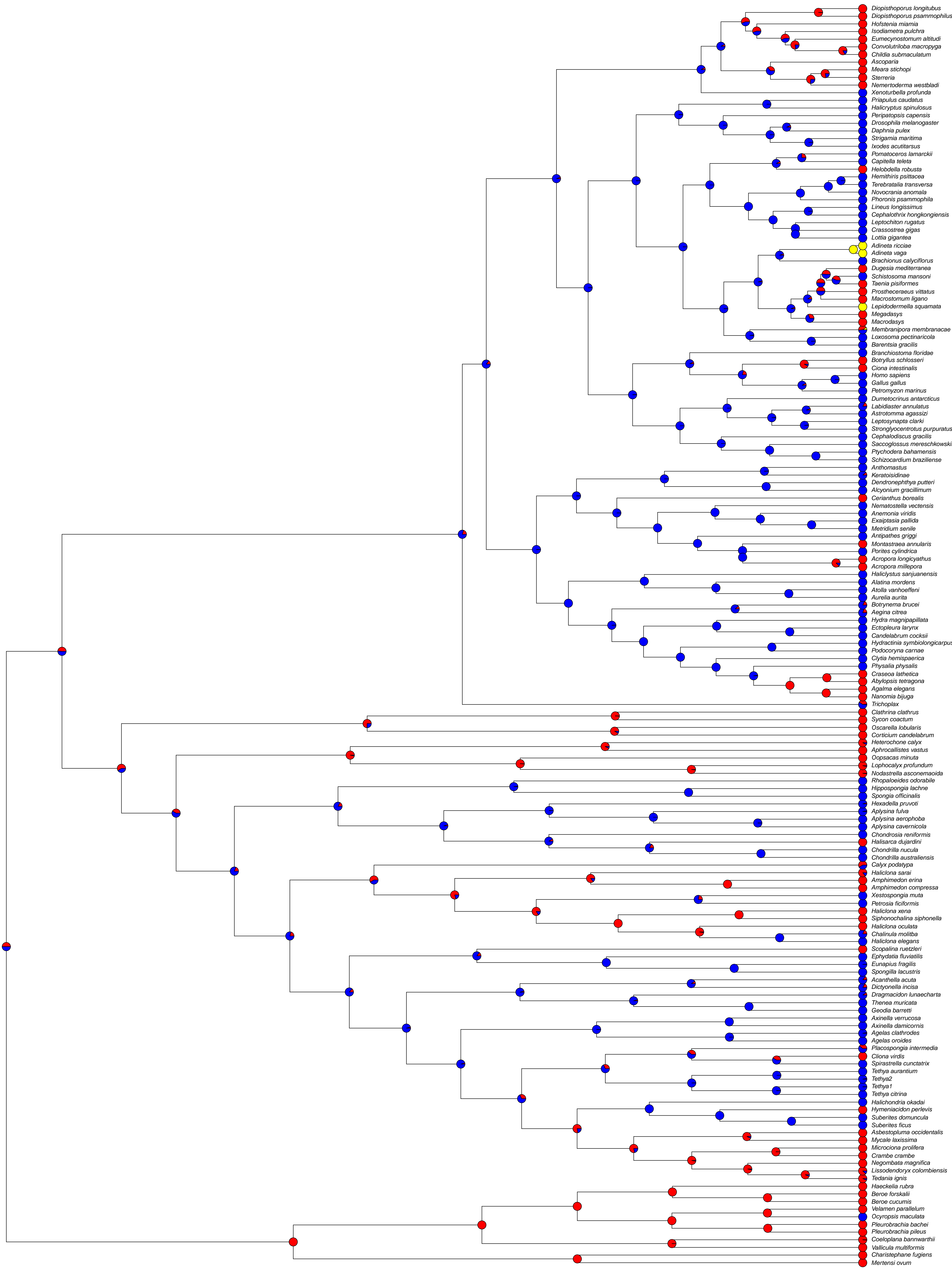

Supplement: Supplementary file 13 — Stochastic character mapping ancestral-state reconstruction for the “ctenophore-sister” topology with the gonochrositc Xenoturbella profunda replacing the hermaphrodite X. bocki. The inclusion of the gonochoristic X. profunda has an especially strong effect on the LCA of Bilateria. Blue circles indicate gonochorism, red circles hermaphroditism, and yellow circles asexuality. (PDF 157 kb) [file 12862_2017_1071_MOESM13_ESM.pdf]

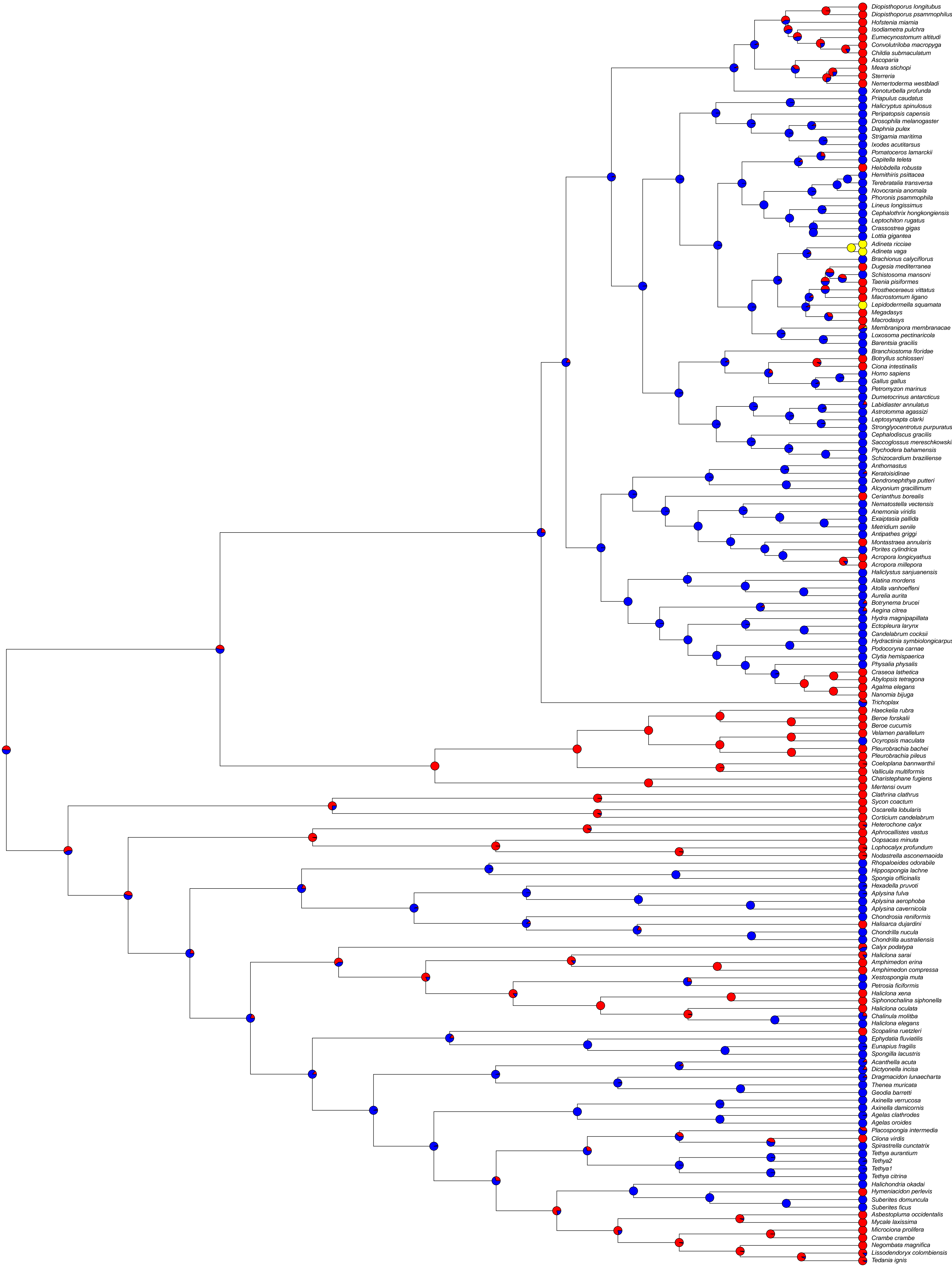

Supplement: Supplementary file 14 — Stochastic character mapping ancestral-state reconstruction for the “sponge-sister” topology with the gonochrositc Xenoturbella profunda replacing the hermaphrodite X. bocki. The inclusion of the gonochoristic X. profunda has an especially strong effect on the LCA of Bilateria. Blue circles indicate gonochorism, red circles hermaphroditism, and yellow circles asexuality. (PDF 157 kb) [file 12862_2017_1071_MOESM14_ESM.pdf]
