# Supplementary material for: A reconstruction of sexual modes throughout animal evolution
Source: BMC Evol Biol. 2017 Dec 6;17:242. doi: 10.1186/s12862-017-1071-3 (PMC5717846; doi:10.1186/s12862-017-1071-3)
Supplement: Supplementary file 8 — Likelihood values from our stochastic character mapping analyses for the predicted sexual mode of the LCA of major animal lineages. (DOCX 18 kb). [file 12862_2017_1071_MOESM8_ESM.docx]

**Additional file 8: Table S2.** Likelihood values from our stochastic character mapping analyses for the predicted sexual mode of the LCA of major animal lineages

|  | Maximum likelihood of  separate sexes/hermaphroditism (%) | |
| --- | --- | --- |
| **Node (LCA of)** | **“ctenophore-sister”** | **“sponge-sister”** |
| Metazoa | 50.7/49.3 | 54.1/45.9 |
| Porifera + Parahoxozoa | 52.1/47.9 | N/A |
| Ctenophora + Parahoxozoa | N/A | 56.3/43.7 |
| Ctenophora | 0/100 | 0/100 |
| Porifera | 43.5/56.5 | 44.2/55.8 |
| Cnidaria + Bilateria | 85.8/14.2 | 85.1/14.9 |
| Cnidaria | 98.9/1.1 | 98.9/1.1 |
| Bilateria | 69.9/30.1 | 74.3/25.7 |
| Xenacoelomorpha | 59.7/40.3 | 65.3/34.7 |
| Ecdysozoa + Spiralia + Deuterostomia | 91.4/8.6 | 92.1/7.9 |
| Deuterostomia | 91.7/8.3 | 92.3/7.7 |
| Ecdysozoa + Spiralia | 99.2/0.8 | 98.8/1.2 |
| Spiralia | 99.7/0.3 | 100/0 |
| Ecdysozoa | 99.5/0.5 | 99.1/0.9 |
